# Pediatric CNS Radiation Oncology: Recent Developments and Novel Techniques

**DOI:** 10.3390/curroncol32030180

**Published:** 2025-03-20

**Authors:** Justin Oh, Samir Patel, Mary-Pat Schlosser, Andrew J. Arifin, Carol Oliveira, Anne-Marie Charpentier, Derek S. Tsang

**Affiliations:** 1 BC Cancer—Vancouver, Vancouver, BC V5Y 4E6, Canada; 2 Cross Cancer Institute, Edmonton, AB T6G 1Z2, Canada; samir.patel2@albertahealthservices.ca; 3 Stollery Children’s Hospital, Edmonton, AB T6G 2B7, Canada; mary.schlosser@albertahealthservices.ca; 4 London Health Sciences Centre, London, ON N6A 5W9, Canada; andrew.arifin@lhsc.on.ca; 5 McGill University Health Centre, Montreal, QC H4A 0B1, Canada; carol.oliveira@mcgill.ca; 6 Centre Hospitalier de l’Université de Montréal, Montréal, QC H2X 3E4, Canada; anne-marie.charpentier.med@ssss.gouv.qc.ca; 7 Princess Margaret Cancer Centre, Toronto, ON M5G 2C4, Canada; derek.tsang@uhn.ca

**Keywords:** pediatric central nervous system (CNS) tumor, pediatric radiotherapy (RT), brain radiotherapy (RT), proton therapy

## Abstract

Radiation therapy (RT) is a cornerstone in the management of pediatric central nervous system (CNS) tumors. Recent advancements in RT delivery and techniques aim to enhance therapeutic effectiveness while minimizing both acute and long-term complications associated with pediatric brain RT. This paper highlights innovative developments in the field, including the clinical indications, benefits, and challenges of proton therapy and stereotactic radiotherapy. The ongoing refinement of risk-adapted RT volumes is highlighted, with examples of newly proposed germinoma RT volumes and hippocampal-sparing RT. Additionally, emerging experimental approaches, including FLASH therapy and theranostics, are also discussed as promising future directions. Further prospective, multi-institutional collaborative studies are essential to validate and expand upon the benefits outlined in this review.

## 1. Introduction

Pediatric radiation oncology has made significant strides over the past few decades, aiming to improve or maintain tumor control while minimizing both acute and late complications associated with radiotherapy (RT). RT plays a crucial role in treating various pediatric central nervous system (CNS) tumors, including primary gliomas, medulloblastomas, ependymomas, atypical teratoid rhabdoid tumor (ATRT), craniopharyngiomas, and germ cell tumors. Depending on the tumor histology and the extent of disease involvement, RT volume can range from focal treatment to craniospinal irradiation that encompasses the entire brain and the spinal thecal sac. However, RT for pediatric brain tumors can lead to permanent and cumulative late side effects, which can lead to lifelong impacts on mood, learning, behavior, social integration, and overall quality of life [1,2,3,4,5,6].

Given that pediatric CNS tumor patients are now living longer due to advancements in multimodal treatments and supportive care, mitigating the risk of late side effects has become an essential aspect of treatment plans [7,8,9]. To optimize the therapeutic ratio of RT for pediatric CNS tumors, innovative techniques and approaches have been developed. Some of these ongoing efforts include the utilization of conformal RT techniques and proton therapy to spare normal brain tissue, de-escalation of RT dose and volume, or omission of RT based on personalized risk factors [9,10,11,12]. This review was conducted to outline some of the most recent advances in the field of radiation therapy, detailing the latest indications, planning techniques, and outcomes of proton therapy; the evolving role of stereotactic radiation therapy (SRT); examples of risk-adopted radiotherapy volumes; experimental therapies on the horizon; and future directions. We conducted a review of the primary literature on the use of proton therapy, SRT, and emerging radiation therapy techniques. The literature from January 2010 to July 2024 was included for the review of contemporary care practices, developments, and challenges. Resources consulted include the Cumulative Index to Nursing and Allied Heath Literature, Medline, Google Scholar, PubMed, and Children Oncology Group (COG) protocols. The following keywords were used for reviewing all sections: children, child, infants, kids, newborns, adolescents, pediatrics, pediatrics, babies, neonates, brain tumors, brain neoplasms, brain cancer, central nervous system, central nervous system tumors, patient outcomes, treatment outcomes, patient care, cognitive, neurocognitive, quality of life, recovery, side effects, and adverse health events. The following keywords were used to narrow down the search results for the respective sections: proton therapy, proton, protons therapeutic use, stereotactic, stereotactic radiotherapy, stereotactic radiosurgery, stereotactic radiation, radiosurgery, hippocampal, hippocampus, FLASH, FLASH-RT, and theragnostic. A glossary of the terms is provided after the conclusion of the article.

## 2. Proton Therapy

Proton beam therapy (PBT) has emerged as a promising alternative to conventional photon therapy in the last few decades. The physical property of a proton particle makes it an appealing RT modality for CNS pediatric tumors. As technology evolves, innovations in PBT delivery continue to enhance the therapeutic ratio, and growing clinical evidence highlights the advantages of PBT. However, challenges remain in optimizing treatment planning, understanding long-term outcomes, and expanding access to PBT globally. The following sections will explore the technical aspects of and recent developments in PBT delivery and planning, as well as expanding clinical applications, benefits, and ongoing challenges.

### 2.1. Planning and Delivery of Proton Therapy

**Figure 1:**
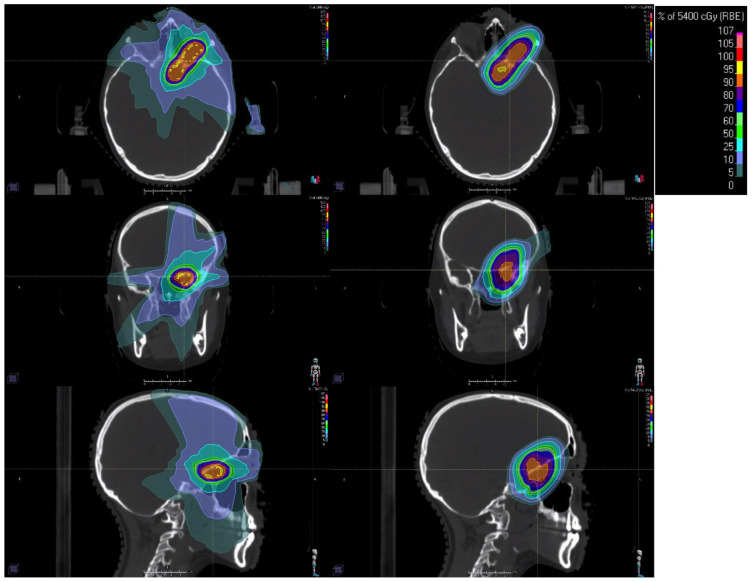
Example radiation dose distributions for a 17-year-old female with an optic pathway glioma. Left column shows a photon intensity-modulated radiotherapy (IMRT) distribution with non-co-planar beams. The right column shows a pencil beam scanning proton beam therapy (PBT) distribution. Isodose line color legend is shown in the top-right inset. The axial, coronal, and sagittal views are shown in the top, middle, and bottom rows, respectively.

Conventional RT with photons, which is widely available around the world, has its peak dose at a shallow depth within the patient, with subsequent attenuation of the beam as it travels through tissue, tumor, and exits the body. Proton beams exhibit a Bragg peak phenomenon, in which energy loss peaks sharply within the tumor before the particle rests, with rapid dose falloff beyond this point. This enables more conformal dose distributions with a reduced dose to the surrounding healthy tissues (Figure 1). Consequently, PBT can lower the risk of acute and long-term side effects compared to conventional photon RT without compromising disease control.

To deliver a particle beam that can treat tumors of several centimeters, the beam must be modified. The two major ways of delivering a proton beam include passive scatter proton therapy (PSPT) and pencil beam scanning (PBS).

PSPT is the standard proton beam delivery method [13]. In PSPT, the narrow proton beam is spread laterally to create a uniform field using scattering systems in which materials of different geometries and compositions scatter the beam homogenously. The beam edges are shaped with patient-specific collimators, and a spinning range modulator wheel varies the beam’s energy over time, creating a spread out Bragg peak. In contrast, magnets are used in PBS to steer the narrow beam in a grid over the treatment volume. The beam’s energy can be modified upstream to allow for intensity-modulated proton therapy (IMPT), thereby improving the conformality of the dose compared to a homogenous passive scatter beam.

Protons spare normal tissues through their sharp dose drop off at the end of their range; range uncertainty can arise from the approximate conversion of CT data into Hounsfield units [14]. This uncertainty can be managed using beam-specific planning target volumes (PTVs), where, in addition to setup and positional uncertainties, the PTV also accounts for proximal and distal range uncertainties [15]. Robust optimization further mitigates setup and range uncertainties by incorporating them into treatment planning optimization algorithms [16].

Most proton centers currently use a constant average RBE factor of 1.1 for PBT when evaluating dose distribution [14]. However, in vitro data have shown that RBE can reach up to 2.0 close to the Bragg peak [17], potentially increasing the risk of adverse events in nearby organs at risk. Many variables influence RBE, though the majority of the variability of RBE can be accounted for by LET [18]. There has been a recent increase in interest in incorporating LET optimization in IMPT planning [19], though, to date, there has not been published clinical data regarding the use of LET optimization and incidence of adverse effects.

Although PBT has been in use for several decades, there remains significant potential for improvement and innovation in its delivery and planning techniques. Proton arc therapy (PAT) is an emerging technique that is not yet commercially available. Akin to volumetric modulated arc therapy (VMAT) with photons, PAT delivers a continuous proton beam along a rotating axis. Limitations to the deployment of PAT have been the computational requirements for PAT planning and the physical limitation of rotating a heavy gantry [20]. One method of mitigating the issues with a rotating gantry is to treat the patient upright and rotate the patient, though this brings up issues of positioning errors and image guidance [21]. Although IMPT can theoretically produce very conformal dose distributions, it is also more sensitive to uncertainties compared to other radiation therapy techniques, which can diminish its effectiveness [14]. The cost and large footprint of PBT equipment limits accessibility; ongoing advances with technology miniaturization are key to lowering the capital cost of building PBT facilities and improving global access. Continued advancements in computational power, machinery, and optimization techniques will be important to advance this technology.

### 2.2. Clinical Indications, Outcomes, and Challenges of Proton Therapy

Given increasing survival post-treatment for pediatric cancers, late effects of treatment are of major concern. PBT is associated with a reduction in acute and long-term toxicities [22,23,24,25,26,27,28], lower rates of radiation-induced second malignancies [29,30,31,32,33,34,35,36], less acute and long-term damage to the developing organs [37,38,39,40,41,42,43], decreased neurocognitive decline [44,45,46,47,48,49,50,51,52,53,54,55], and long-term health-related quality of life reported as commensurate with healthy children [56]. Systematic and meta-analysis study of neurocognitive testing results demonstrated significantly better outcomes for the proton cohort compared to the photon cohort, including full-scale intelligence quotient (IQ) (Z-score 0.75, *p* < 0.001), verbal comprehension index (Z-score 0.46, *p* = 0.001), perceptual reasoning index (Z-score 0.69, *p* < 0.001), and working memory index (Z-score 0.35, *p* = 0.016) [47]. This makes PBT the preferred modality for radiation treatment in many pediatric and adolescent patients. This is especially the case for pediatric and adolescent patients with CNS tumors, where sparing large volumes of the brain from a radiation dose aims to preserve neurocognitive function and reduce long-term side effects.

Recent studies on PBT for CNS tumors have reported favorable and comparable outcomes to photon RT, as well as low toxicity [57,58,59,60]. Studies comparing PBT in ependymoma have shown comparable outcomes to photon RT without unexpected toxicity and no difference in brainstem toxicity [61,62,63]. In medulloblastoma, reports show improved overall and recurrence-free survival with PBT versus a radiation-sparing approach in young children and favorable acute and preliminary long-term toxicity [64,65,66]. A systematic review of RT modalities for medulloblastoma confirms PBT as a favored treatment modality over photons for children with this disease [67]. For craniopharyngioma, surgery and PBT are reported to have comparable survival and complications with improved cognitive outcomes and excellent disease control with rare acute toxicity [68,69]. When used for low-grade glioma, PBT was associated with a reduced dose to developing tissue, diminishing its acute toxicities without compromising disease control and, in unresectable spinal low-grade glioma, long-term disease control with limited toxicity [70,71].

Radionecrosis and brainstem injury are rare complications of both PBT and photon RT. While there have been some concerns that brain radionecrosis risk increases with the use of PBT due to increased relative biological effectiveness (RBE) and linear energy transfer (LET) [72,73,74,75], other reports have suggested no significant difference between the two modalities [76,77,78]. Given that the majority of pediatric brain tumors treated with RT are infratentorial tumors, a brainstem injury is a potentially serious complication. Recent data demonstrate that the risk of grade 3 or higher injury is low and may be comparable to photon therapy, and that risk may correlate with the dose exposure to the brainstem (Table 1).

Treatment planning that adjusts RT plans based on RBE and linear energy transfer LET, discussed in the previous section, may further help mitigate these and other adverse effects of treatment [75,85]. Advanced imaging modalities, including positron emission tomography and magnetoencephalography, have been used to assess brain injury after radiotherapy or trauma in children [86,87]. Further research is essential to more clearly define the role of advanced imaging in enhancing the quality of RT. This includes potential applications in functional contouring, adaptive planning, and the post-treatment surveillance of patients.

Access to PBT is limited in certain countries. Internationally, there were 120 PBT facilities in clinical operation, including 48 in the United States, as of June 2024, with an additional 30 facilities under construction as of October 2024 [88]. Clinical practice guidelines for appropriate referral for patients out of country for PBT have been developed in several jurisdictions without locally available PBT facilities [89,90,91,92,93]. Primarily, referral criteria include an indication of curative intent RT and reasonable expectations for clinically meaningful reduction in toxicity, which may include assessments by comparative dosimetry or normal tissue complication probability modeling between PBT and photon RT plans. Centers without proton therapy in countries such as Canada and Australia are developing dedicated funding and clinical care pathways to reduce the time interval from diagnosis to treatment.

There is a need for better quality evidence to guide the use of PBT in pediatric CNS tumors. Randomized controlled trials (RCTs) of PBT versus photon RT have not been conducted in children with brain tumors, and many would consider a RCT to be unethical due to the lack of clinical equipoise in this setting [94]. Many publications have relied on non-randomized study designs, including prospective and retrospective case series and small cohorts, largely due to the rarity of pediatric CNS tumors, inconsistent assessment criteria, and limited follow-up durations. Within these research limitations, prospective, multicenter registry studies databases tracking treatment parameters and clinical outcomes can be helpful. The Pediatric Proton/Photon Consortium Registry aims to standardize prospective data collection for outcomes analysis across 24 institutions in the United States, Canada, and Australia [95]. A European pediatric registry by Harmonic in the European Union could enable transatlantic data matching to further improve the quality of the available evidence [96].

## 3. Stereotactic Radiotherapy for Pediatric Brain Tumors

Stereotactic radiotherapy, including stereotactic radiosurgery (SRS) and fractionated stereotactic radiotherapy (FSRT), is a highly precise radiation technique used to treat brain tumors while minimizing damage to healthy tissues. By utilizing advanced imaging, immobilization systems, and specialized radiation equipment, these techniques allow for targeted treatment in both pediatric and adult patients. While widely used in adult oncology, their role in pediatric brain tumors is still being explored, particularly for recurrent or residual disease. The following sections will discuss the planning and delivery of stereotactic radiotherapy, as well as its clinical applications, outcomes, and challenges in pediatric patients.

### 3.1. Planning and Delivery of Stereotactic Radiotherapy

Fractionated stereotactic radiotherapy (FSRT) and stereotactic radiosurgery (SRS) are radiation techniques that are typically used to treat primary or metastatic brain tumors with or without prior surgery. While they both grant a precise delivery of high-dose ionizing radiation, in FSRT, the dose is divided into 3 to 30 fractions, while SRS typically refers to single-fraction regimens. Stereotactic radiotherapy requires accurate localization of the lesion using high-resolution imaging such as thin-slice volumetric MRI with contrast, rigid immobilization with an invasive head frame or dedicated frameless system, small-field dosimetry, and accurate image guidance delivery [97,98]. The radiation regimen and total dose are based on tumor size and location and should take patient-specific factors such as histology into account [99]. While both photons and protons can be utilized, photon SRS and FSRT are used much more widely due to accessibility, robustness of evidence, and caution about proton dose uncertainty while employing a high dose for a small target and the potential limitation of beam angles with immobilization devices [100].

For both treatment modalities, precise technical execution is crucial to ensure safety and efficacy [98,101]. Technical requirements include patient immobilization to minimize patient movement during treatment (e.g., custom masks or frames), high-resolution imaging to allow for precise delineation of the target and critical structures, advanced treatment planning systems to create highly conformal dose distributions, and rigorous quality assurance procedures to verify the accuracy of the treatment delivery (e.g., regular equipment calibration, verification of treatment plans, and peer review) [101]. These techniques appear particularly valuable in children with brain tumors due to their high conformality and ability to avoid healthy tissues, thereby reducing the impact of cranial irradiation on neurocognition [102,103], hearing [104], growth [105], neuroendocrine, and neurovascular outcomes [106], as well reducing the risk for secondary neoplasms [102,104]. The evidence supporting these techniques within the pediatric population is evolving but continues to be limited to case series and retrospective studies [107]. However, with the improvement of radiosurgery units and stereotactic delivery systems, as well as accumulating evidence supporting FSRT and SRS in pediatric patients with CNS tumors, an increasing number of children, particularly in the setting of recurrence, are being treated with FSRT or SRS during their cancer journey.

There are different RT machines capable of SRS or FSRT in the context of pediatric CNS tumors, broadly divided into dedicated or non-dedicated units. The two main dedicated devices are Gamma Knife^®^ and CyberKnife^®^. Gamma Knife^®^ was first installed in Stockholm by Professor Leksell in 1968. Multiple iterations of the prototype have since been developed but always rely on multiple cobalt-60 sources producing a narrow beam of gamma radiation, all directed at a single point in space. Different collimator sizes are used to create treatment volumes of 4–18 mm [108]. Nonspherical targets may be treated by overlapping different collimator sizes. The Leksell Gamma Knife^®^ has traditionally relied on an invasive stereotactic head frame pinned onto the patient’s head to achieve a target accuracy of less than 1 mm [109]. Due to the immaturity of the skulls of young children, complications such as penetration of the cranial vault by a fixation pin of the stereotactic frame have been reported [110]. Newer versions of the Gamma Knife^®^ allow non-invasive frameless treatment with the use of a relocatable face mask, which may be more appropriate for the pediatric population. Similarly, CyberKnife^®^ has the advantage of being non-invasive (frameless) and has the unique feature of being composed of a linear accelerator (LINAC) directly mounted on a robotic arm. It allows for SRS delivery, as well as stereotactic body radiation therapy (SBRT). In addition to CyberKnife^®^ and Gamma Knife^®^, ZAP-X is a relatively new LINAC-based, dedicated SRS device that has been used for treating brain metastases, gliomas, and benign intracranial tumors [111,112,113,114]. It is a self-contained system that operates with low-energy radiation and does not require dedicated radiation bunkers [111]. While preliminary comparisons with other SRS machines appear promising, no pediatric cases have been treated to date and thus fall beyond the scope of this review.

The access to SRS for pediatric cancer patients may be facilitated by using non-dedicated units, such as newer generations of LINAC, which may deliver SRS and SBRT in addition to VMAT.

### 3.2. Clinical Indications, Outcomes, and Challenges of Stereotactic Radiotherapy

Stereotactic radiosurgery applications usually follow one of the following three clinical scenarios: (1) to treat a tumor recurrence after prior RT, (2) to escalate the dose to the residual tumor after a conventional RT treatment, and (3) to give a large single dose of radiation for a low-grade tumor.

#### 3.2.1. Ependymoma

The role of SRS for ependymoma is mainly in the recurrent setting, after initial surgical resection and RT. The International Gamma Knife Research Foundation published a multicenter study evaluating the role of SRS for recurrent intracranial ependymomas in 2019 [115]. Their series comprised both pediatric and adult patients, with a median age of 16.3 years. The median dose to the tumor margin was 15 Gy, resulting in a 3-year progression-free survival (PFS) of 56%. Radiation was well tolerated, with 8% of their patients experiencing symptomatic adverse effects from RT, all controlled with oral corticosteroids. Stanford University recently reported their series of pediatric and adult patients receiving SRS for ependymoma between 1998 and 2023 [116]. Fourteen patients were pediatric. The median prescribed dose was 16.6 Gy at the 77% isodose line. For the children, they noted a 59.6% tumor control rate at 5 years. Their report did not detail prior radiation therapy received but stated a median interval of 69 months between diagnosis and SRS. For recurrent ependymoma, the selection of SRS over conventional fractionated for reirradiation also depends on the type of relapse. In the updated series from St. Jude Children’s Research Hospital [117], patients presenting with a distant-only failure then treated with salvage craniospinal irradiation had the best 5-year survival (76%). Only 1 patient in that series of 101 pediatric patients received SRS as part of their reirradiation, and it was given in conjunction with photon RT. In comparison, it was used for 4 of the 14 patients who received a third course of RT.

#### 3.2.2. Medulloblastoma

For medulloblastoma, similar indications for SRS have been described: recurrence, metastatic disease, and upfront boost. For patients with a recurrent localized medulloblastoma, Saran et al. published their experience of hypofractionated stereotactic radiosurgery (30–40 Gy in 6–8 fractions) [118]. Twelve patients with recurrent medulloblastomas and two patients with residual supratentorial PNET constituted the cohort. Their PFS at 1 and 3 years was 80% and 48%, respectively. One of the largest series on SRS or FSRT for medulloblastoma was published in 2006 by a group from Japan [119]. Eighteen lesions in twelve patients were treated, either in a single session (*n* = 8) or with a fractionated regimen (*n* = 10). The tumor response rate to radiation was high, with 14 lesions out of 18 disappearing within 1–6 months after treatment. Progression of the disease remained a concern for patients treated for metastasis, as the eight patients in this subgroup had disease progression outside of the treated volume. Using SRS to administer a boost of 4.5–10.0 Gy during the primary treatment for medulloblastoma in children was proposed by Woo et al., 1997 [120], with no occurrence of radionecrosis in a very limited number of patients (*n* = 4).

#### 3.2.3. Glioma

Radiosurgery for gliomas is most often used in low-grade disease, either to treat unresectable masses as an adjuvant treatment after subtotal resection or in the recurrence setting. Early on in 1996, Somaza et al. published their report on nine pediatric patients with juvenile pilocytic gliomas treated with SRS after subtotal resection (*n* = 7) or biopsy (*n* = 2) [121]. Two patients had previously failed fractionated RT. The mean marginal dose was 15 Gy (range 12–18 Gy) to a mean target volume of 4.2 cm^3^, resulting in a 100% control rate. No morbidity or mortality due to SRS was observed. In 2001, an Austrian group reported their experience with Gamma Knife^®^ radiosurgery for deep-seated brain tumors in 50 children [102]. Of these, 12 children aged 5 to 15 years had low-grade tumors that were treated with radiosurgery, 10 of which had subtotal resection and 2 had stereotactic biopsy only. The treatment volume ranged between 0.5 and 15.3 cm^3^. The marginal dose ranged from 15 to 18 Gy to a mean isodose line of 50%, resulting in two cases with stable disease, two with progressive disease, and five with tumor shrinkage. Three patients were lost to follow-up. Only one child developed edema necessitating steroid treatment. The Karolinska Hospital in Sweden reported on 19 children with pilocytic gliomas treated with Gamma Knife^®^ radiosurgery, of which 16 had prior subtotal resection [122]. The median tumor size treated was 3.3 cm with a median dose of 11.3 Gy (9–20 Gy), resulting in a 100% control rate. Increased enhancement or edema were observed in 25% of cases, and two patients developed cysts. In 2009, Kano et al. reported on 50 pediatric patients with juvenile pilocytic astrocytoma treated with Gamma Knife^®^ radiosurgery between 1987 and 2006 [123]. Three patients had previous fractionated RT and two had RT and chemotherapy. The median target volume was 2.1 cm^3^ (range 0.17–14.4 cm^3^), which were treated with a median margin dose of 14.5 Gy (11–22.5 Gy). They reported PFS of 91.7%, 82.8%, and 70.8% at 1, 3, and 5 years, respectively. Adverse radiation effects were seen in five patients (10%), of which three were symptomatic due to peritumoral edema. Most recently, Weintraub et al. published their experience with 24 patients aged 4–18 years with pilocytic astrocytoma and treated with SRS [124]. The mean tumor volume was 2.4 cm^3^. The lesions were treated with a median marginal dose of 15 Gy. Treatment response was seen in 75%, with complete tumor resolution being achieved in 21%. Progression was documented in 17% of the cases. They found a significant association between greater tumor volumes and disease progression (4.25 versus 2.0 cm^3^, *p* < 0.001). Peritumoral edema was reported for 12.5% of patients. For high-grade gliomas, the role of SRS/FSRT is less well defined for the pediatric population. One small series reported on seven pediatric patients aged 2.6 to 7.7 years with diffuse intrinsic pontine gliomas (DIPG) treated primarily with 25 Gy in five fractions [125]. Three patients developed symptomatic tumor necrosis treated with steroids and bevacizumab. The literature on adult patients suggests improved survival with a more favorable safety profile of SRS than repeat surgical resection in progressive, high-grade gliomas [126].

#### 3.2.4. Craniopharyngioma

Craniopharyngiomas are benign tumors of the suprasellar region that can be approached with either gross total resection or with a more limited resection followed by RT with similar tumor control rates [127,128,129]. Controversies remain regarding the optimal approach and timing of RT [130], particularly because of the inherent morbidity associated with treatment in this location: visual loss, endocrine deficits, hypothalamic syndrome, and neurocognitive disorders. Recent publications are mainly investigating the role of PBT for this benign tumor [68,131]. However, small, well-circumscribed tumors that are localized at a certain distance from the optic pathway may be considered for SRS. In 2020, a Japanese multi-institutional study gathered data from 242 patients (adults and children) with craniopharyngioma treated with Gamma Knife^®^ [132]. The mean target volume was 3.1 mL, and the mean marginal dose was 11.4 Gy. The five- and ten-year PFS were 62.2% and 42.6%, respectively, with a 6.2% complications rate. Another large series of patients treated with Gamma Knife^®^ for residual or recurrent craniopharyngioma was published by a group from Taipei, consisting of 137 patients aged 1.5–84.9 years [133]. PFS at 5 and 10 years was 77.3% and 61.5%, respectively. Other groups have reported on control rates of 75–100% after FSRT to 50–55 Gy [134,135,136].

#### 3.2.5. Brain Metastases

Despite the frequent use of SRS for adult cancer patients with a limited number of brain metastases [137,138,139], few studies have addressed this indication in the pediatric population. In SRS for brain metastases, lower doses and/or increased fractionation are recommended with the increasing size of lesions due to the higher risk of radionecrosis of larger lesions [99]. In the literature on adults, the 1-year local control rates for brain metastases are >85% and >95% for up to 20 mm lesions treated with 18 Gy and 24 Gy, respectively, and approximately 70% and 75% for lesions >20 mm with the usual doses of 15 to 18 Gy [140]. FSRT should therefore be considered for larger lesions to reach comparable local control rates to smaller lesions [140]. The most recent guidelines allow for treatment of up to 10 brain lesions, assuming a functional performance status of the Eastern Cooperative Oncology Group (ECOG) of 0–2 [139]. In the pediatric population, the M.D. Anderson Cancer Centre reported its experience with SRS for brain metastases from a solid tumor in 54 pediatric patients in 2014 [141]. Most patients in their cohort had a single brain lesion (60%) and metastasis from a sarcoma (54%). They did not use a uniform treatment approach, with some patients having a resection (25%) while others received whole-brain RT (10%), SRS (4%), chemotherapy (8%), or a combination of treatments. Overall, only 13% of their cohort received SRS for their brain metastasis. However, patients treated for their brain metastases had significantly improved overall survival (0.9 months versus 8 months). More recently, the Dana-Farber Cancer Institute in Boston published their cohort of 26 pediatric patients with solid tumor CNS metastases [142]. Ten of their patients received focal RT; six of them were treated with SRS/SFRT. Stereotactic treatment regimens consisted of 18 Gy/1 fraction, 20 Gy/1 fractions, 25 Gy/5 fractions (including 1 with a simultaneously integrated boost to 30 Gy), and 30 Gy/5 fractions. The overall survival of patients receiving focal RT was 12.8 months with a CNS PFS of 4.6 months. Half of the patients who were initially treated with focal RT went on to receive additional cranial RT.

Despite advances in technology, clinical data on the efficacy and toxicity of SRS and FSRT for pediatric brain tumors remain limited to institutional series. The International Stereotactic Radiosurgery Society recently published an exhaustive meta-analysis on pediatric cranial stereotactic radiosurgery [107]. Their work summarizes the published data for SRS and FSRT in medulloblastomas, craniopharyngiomas, ependymomas, and gliomas. The group further provides practice guidelines for pediatric cranial radiosurgery. Hopefully, similar collaborations in the future might generate a better understanding of the optimal use of SRS and FSRT for children with brain cancers.

## 4. Recent Developments in Pediatric CNS Radiotherapy Volumes

Decision-making surrounding radiotherapy targets and elective volumes are key components determining the success of a course of treatment. Research in pediatric neuro-oncology seeks to optimize the balance—and sometimes conflict—between larger volumes and comprehensive radiotherapy, which may improve disease control, versus a prevailing desire to minimize radiotherapy target volumes to reduce cognitive sequelae. In this section, we present a critical discussion of this balance using two scenarios: intracranial germ cell tumors (GCTs) and tumors requiring whole brain/craniospinal irradiation. For GCTs, treatment volumes have evolved over time to strike a balance between long-term toxicities and achieving tumor control. For patients requiring whole brain/craniospinal irradiation, we will discuss whether therapeutic de-escalation via hippocampal avoidance is a viable treatment worthy of study and future implementation.

### 4.1. CNS Germ Cell Tumors

Treatment for children with intracranial germ cell tumors has evolved over the past decades. For patients with germinomas, there has been a new focus on combining chemotherapy with RT, which permits a decrease in the intensity and treatment volume of RT while maintaining very high tumor control probability. In contrast, non-germinomatous germ cell tumors (NGGCTs) have been the subject of practice variation globally; contemporary approaches have involved applying novel RT volumes to maximize disease control while minimizing undue toxicity. In this section, we will provide an overview of radiation treatment for both germinomas and NGGCTs.

#### 4.1.1. Germinoma

Intracranial germinoma typically occurs in the sellar and suprasellar regions and is frequently associated with diabetes insipidus. Diagnosis is by tumor markers alone (undetectable or isolated mild elevated beta-HCG in the serum or CSF) with or without biopsy. Historical treatment was with RT alone, and this continues to be applied in some jurisdictions around the world. When treating with RT alone, a curative approach necessitates craniospinal irradiation (CSI) to a dose of 25–30 Gy, with a focal tumor boost to 30–50 Gy [143]. More modern approaches de-escalate RT to 18 Gy CSI, with a focal tumor boost to 36 Gy [144].

**Figure 2:**
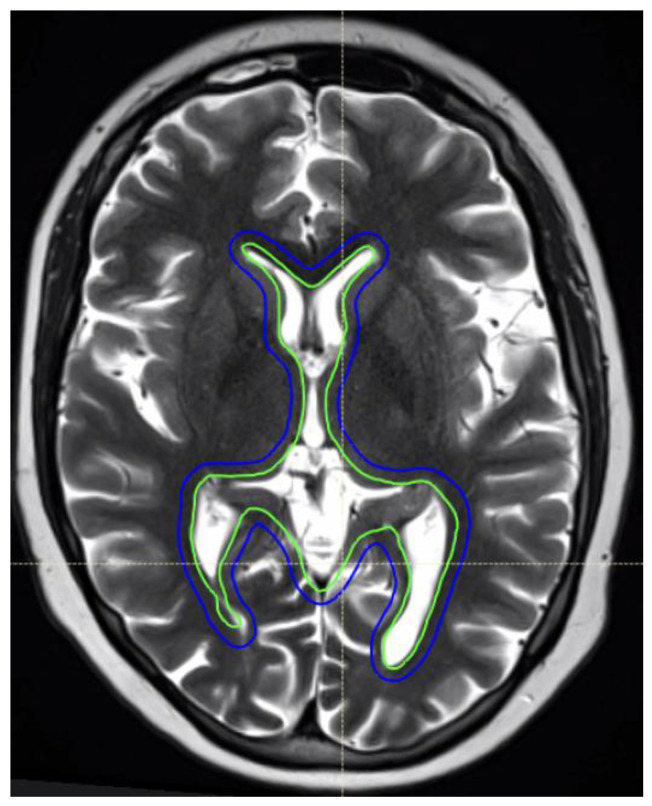
An 11-year-old female patient with intracranial germinoma. The intracranial RT target volumes are shown as follows: whole ventricular RT CTV (green) and 3 mm PTV geometric expansion (blue).

The Children’s Oncology Group (COG, ACNS1123 stratum 2) evaluated the combination of chemotherapy (carboplatin and etoposide) and whole ventricular radiotherapy (WVRT) to 18–24 Gy, followed by a focal tumor boost to 30–36 Gy [12]. The tumor control outcomes were excellent, with 3-year PFS of 94–95%. The addition of chemotherapy replaces CSI and its associated late toxicities; WVRT (Figure 2) substantially reduces the volume of brain irradiated compared to CSI. Additionally, there are some data suggesting that a focal RT boost after ventricular RT may not be necessary, and patients may be adequately treated with ventricular RT alone [145]. In addition, RT de-escalation to specific regions of the ventricles may be possible with photon RT while maintaining tumor control [146]. The COG is now conducting a next-generation germinoma study, ACNS2321 (NCT06368817), which is prospectively evaluating reduction in ventricular RT doses to 12–18 Gy, followed by a focal tumor bed boost to 24–30 Gy.

#### 4.1.2. Non-Germinomatous Germ Cell Tumor

Patients with NGGCTs have poorer outcomes as compared to intracranial germinomas. Tumor markers (AFP and/or beta-HCG) are typically elevated, with the histological subtype confirmed by biopsy or resection. Chemotherapy is required; the COG uses carboplatin/etoposide alternating with ifosfamide/etoposide for six cycles, followed by CNS-directed radiation [147].

For children with localized non-germinomatous germ cell tumors, focal RT is a treatment option but leads to inferior tumor control. In the SIOP-CNS-GCT-96 study, the 5-year PFS was 72% with focal RT [148]. The COG evaluated CSI 36 Gy, followed by a focal boost to 54 Gy; this intensive regimen led to excellent tumor control. In ACNS0122, the 5-year PFS was 92% in patients with complete or partial response to induction chemotherapy and 84% among all patients regardless of response [149]. However, this RT regimen—especially 36 Gy CSI—puts patients at a substantial risk of long-term side effects.

The subsequent COG germinoma study, ACNS1123, evaluated ventricular RT 30.6 Gy, with a focal RT boost to 54 Gy. This therapeutic de-escalation study, which reduced the RT volume from CSI to WVRT and reduced the initial RT dose from 36 Gy to 30.6 Gy, led to very good tumor control outcomes, with a 3-year PFS of 88% (for patients eligible for protocol-specified de-escalation) [147]. However, a small number of patients had distant spinal recurrences, which were partially due to some children without normalization of serum markers remaining in the study and incorrectly receiving de-escalated therapy [147]. To address the issue of out-of-field spinal failures, the currently open ACNS2021 study is evaluating ventricular and spinal canal RT to 30.6 Gy, followed by a focal tumor boost to 54 Gy (NCT04684368). PBT is encouraged for patients in the study to minimize RT exposure to normal tissues in the brain [37] and spine. A summary of selected NGGCT studies is presented in Table 2.

### 4.2. Hippocampal-Sparing Technique

**Figure 3:**
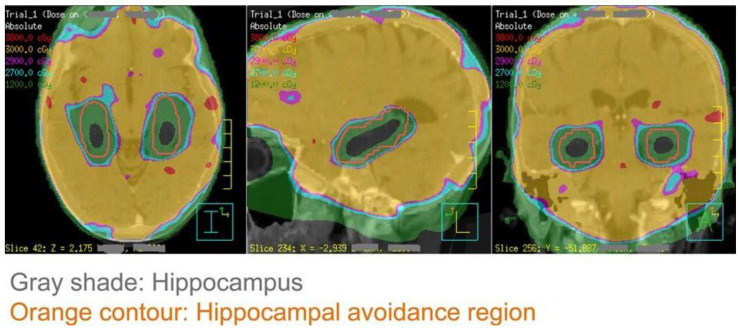
An example of the hippocampal−sparing whole brain radiotherapy plan. Grey shade represents the hippocampus, which is spared from a full dose of radiotherapy [154].

Intracranial radiotherapy can have a significant impact on neurocognitive functions, particularly memory formation and learning. Memory formation is a complex and dynamic process involving multiple anatomic compartments of the brain, with the hippocampus being one of the critical structures responsible for this function [150]. Basic science studies have demonstrated that radiation exposure can lead to a decline in neural stem cells and increased inflammation in the hippocampal region [151,152]. Observational and single-arm prospective studies have further shown a correlation between the mean radiation dose to the hippocampus and the development of short-term memory and learning impairments in adults [153]. The hippocampal avoidance (HA) RT technique, designed to minimize radiation exposure to the hippocampi while ensuring adequate coverage of the remaining brain, has been proposed as a strategy to mitigate the risk of neurocognitive decline (Figure 3).

A prospective randomized trial demonstrated that, in adult patients receiving whole-brain radiotherapy (WBRT) for brain metastases, HA techniques that reduce the mean hippocampal dose result in significantly less deterioration in executive function, learning, and memory, without compromising tumor control [155].

In the pediatric CNS setting, there have been no prospective randomized trials to establish the effectiveness and safety of hippocampal avoidance (HA) RT. However, several pediatric studies have demonstrated a correlation between the RT dose to the hippocampi and memory outcomes. Zureick et al. found that children treated with PBT for primary brain tumors who received higher doses to the left hippocampus experienced worse visual and verbal memory outcomes [156]. Another study from the Hospital for Sick Children reported associations between hippocampus dose and verbal comprehension across different types of pediatric brain tumors treated with RT [157]. Similarly, Acharya et al. demonstrated that, for the children and young adolescents treated with RT for low-grade glioma (LGG), a higher hippocampal dose was associated with worse neurocognitive outcomes [158]. Overall, the most recent literature suggests that reducing the mean dose and volume of both the right and left hippocampus to 20–40 Gy may translate to better cognitive preservation. A summary of select literature on the hippocampus dosimetry correlation with the cognitive outcome is outlined in Table 3.

While hippocampal-sparing is considered a valuable approach to mitigating neurocognitive decline, it carries a potential risk of disease relapse due to insufficient RT doses within and around the hippocampi. Ideal candidates for HA RT would be patients with localized and curable tumors which oncologic outcomes would not be compromised by sparing the hippocampi. Such tumors include low-grade glioma, craniopharyngioma, and ependymoma, which typically exhibit favorable local control and a low risk of out-of-field relapses [162,163,164,165].

Exploratory studies have highlighted the technical feasibility of HA RT for medulloblastomas as well. The standard treatment for MB involves craniospinal irradiation (CSI), targeting the entire brain and spinal canal due to their risk of relapse. Baliga et al. reported in a single-institution series that, among 25 patients with relapsed MB who underwent detailed magnetic resonance imaging (MRI), none experienced relapse within the hippocampus [166]. However, 2 out of 25 patients (8%) had relapses in peri-hippocampal regions (0–5 mm from the hippocampi), both of whom also exhibited leptomeningeal relapse. The authors concluded that the rarity of isolated hippocampal relapses supports the further exploration of HA techniques for MB. Similarly, Padovani et al. demonstrated that high-risk MB patients without upfront metastasis treated with 36 Gy of CSI did not develop metastasis within or near the hippocampi [167]. Conversely, patients with upfront metastasis exhibited a high risk of subsequent metastatic relapses, including relapses within 15 mm of the hippocampi (43%). These findings suggest that patients with localized, molecularly lower-risk disease may be optimal candidates for HA-CSI consideration.

Overall, studies indicate that HA-sparing techniques warrant exploration in patients with localized primary brain tumors and favorable long-term prognoses, such as low-grade glioma, craniopharyngioma, ependymoma, and some children with medulloblastomas. Future research should focus on defining hippocampal dose constraints and validating neurocognitive and quality-of-life metrics tailored for pediatric patients undergoing cranial RT. Additionally, prospective, multi-institutional studies are crucial to standardize the implementation of HA-sparing techniques and ensure consistent practice without compromising tumor control.

## 5. Experimental Radiation Therapies on the Horizon

### 5.1. FLASH Therapy

Many innovative experimental radiation approaches are being developed in the pediatric CNS realm. One emerging novel RT delivery is FLASH-RT, which utilizes a very high dose rate (>40 Gy/second) of radiation that aims to reduce treatment time and minimize side effects while maintaining effective tumor control [168]. Although the exact mechanisms underlying normal tissue preservation are still under investigation, preclinical studies specific to brain RT have shown that FLASH-RT may preserve neurocognitive function by protecting neuronal dendritic spines, reducing neuroinflammation, and decreasing reactive oxygen species compared to conventional RT [169,170,171,172,173]. A small prospective trial demonstrated the feasibility of FLASH-RT for treating extremity bone metastases in adult patients, and another trial is currently underway to evaluate its use for thoracic bone metastases in adults [174,175]. Given these findings, there is growing interest in applying the FLASH-RT technique to pediatric CNS tumors to achieve effective tumor control while minimizing both acute and long-term side effects, particularly neurocognitive decline [176,177,178]. However, significant limitations remain, including the need to further elucidate the mechanisms of neurocognitive preservation in vivo, establish appropriate dose and constraint parameters, and improve dose penetration for deeper tumors [176].

### 5.2. Theranostics

Theranostics also holds promise for pediatric CNS tumors. The term theranostics combines diagnostics and therapy, referring to the use of targeted molecules paired with radioactive tracers for both diagnostic imaging and therapeutic purposes [179]. In pediatric oncology, a well-established example is the use of metaiodobenzylguanidine (MIBG) combined with iodine-131 (I-131) to treat neuroblastoma [180]. MIBG, a norepinephrine analog, is selectively taken up by neuroblastoma cells, allowing the attached I-131 to emit cytotoxic beta particles [180]. MIBG-I-131 therapy with or without other systemic agents has been studied in high-risk and relapsed or refractory neuroblastomas, with promising results [181]. Emerging theragnostic approaches in pediatric CNS tumors include phase 1 studies evaluating the combination of lutetium beta-emitter and somatostatin analogs for embryonal tumors, as well as 8H9 monoclonal antibodies conjugated with iodine-124 (I-124) for high-grade gliomas delivered via convection-enhanced delivery [182,183]. Despite these advances, several challenges remain, including identifying the safest and most effective routes of administration, understanding the safety profiles of theragnostic agents in pediatric populations, and addressing the limited availability of radioisotopes, which are produced in only a few specialized facilities. To fully establish the role of theragnostic agents in pediatric CNS tumors, large-scale prospective phase 2 and phase 3 trials are needed to validate their effectiveness and safety.

## 6. Conclusions

The management of pediatric central nervous system (CNS) tumors demands complex multidisciplinary collaboration. RT remains a cornerstone of treatment, with advancements focused on optimizing oncologic outcomes while minimizing both acute and long-term toxicities. Improvements in proton therapy planning and delivery, such as intensity-modulated proton therapy (IMPT), proton arc therapy (PAT), and the incorporation of linear energy transfer (LET) in planning, may continue to enhance therapeutic benefits and expand clinical applications. Stereotactic techniques show promise, particularly for small and recurrent tumors, though further prospective studies are needed to validate the outcomes, complications, and patient selection criteria. Treatment volume de-escalation in the form of risk-adopted radiotherapy volumes in germinomas or hippocampal-sparing strategy in primary brain tumors such as gliomas, craniopharyngiomas, ependymomas, and medulloblastomas may be feasible and should be investigated in future studies. Emerging modalities such as FLASH-RT and theragnostic agents offer promising preliminary results but require further large-scale studies and clinical trials to establish their safety and efficacy. The integration of innovative technologies with evidence-based practices holds great promise for improving outcomes in pediatric CNS tumor management.

## Figures and Tables

**Table 1:** Summary of the literature on brainstem injury risk with photon and proton therapy in the last 10 years.

Author and Study Type	Photon/ Proton	Patient Population	Median Follow Up	Treatment Dose	Injury Rate	Identified Risk Factors
Indelicanto et al., 2014 [74] Single institution retrospective	Proton	313 children Median age 5.9 years All brain tumors	2 years	Median prescribed dose 54 Gy	2-year cumulative brainstem toxicity 3.8% 2-year grade 3+ toxicity 2.1%	V55 and Dmax to brainstem
Giantsoudi et al., 2016 [79] Single-institution retrospective	Proton	111 children Median age 7 years Medulloblastoma	4.2 years	Median CSI 23.4 Gy, boost 54 Gy	3.6% for any grade, 2.7% for grade 3+ at 5 years	N/A
Gentile et al., 2018 [76] Single-institution retrospective	Proton	216 children Median age 6.6 years IF tumors	4.2 years	Median dose 54 Gy Median brainstem Dmax 53.6 Gy	2% for any grade at 5 years	Higher brainstem Dmax and V55
Haas-Kogan et al., 2018 [75] Multi-institutional retrospective	Proton	671 children Median age 5.4 years IF tumors	3 years	Variable—54–59.4 Gy depending on institution and histology	2.4% for grade 2+ necrosis, 1.3% for grade 3+ Fatal brainstem injury 0.4%	Higher prescription dose and higher doses to the brainstem
Vogel et al., 2019 [80] Single-institution retrospective	Proton	166 children Median age 10 years All brain tumors, 50% IF	19.6 months	Median dose 54 Gy Median brainstem Dmax 55.4 Gy	0.7% symptomatic necrosis	N/A
Baliga et al., 2022 [81] Single-institution retrospective	Proton	178 children Median age 8.1 years Medulloblastoma	9.3 years	18–36 Gy CSI, boost to cavity or posterior fossa 54–55.8 Gy	1.9% at 10 years	N/A
Upadhyay et al., 2022 [82] Single-institution retrospective	Proton	595 children Median age 6.3 years All brain tumors, 76% IF	39.6 months	Median prescribed dose 54 Gy	3.2% symptomatic brain injury	Higher V50–52 to brainstem, female gender, pre-RT high dose chemotherapy, lack of CSI
Gunther et al., 2015 [83] Single-institution retrospective	Proton	37 children Median age 4.4 years Ependymoma	40.6 months	Median dose 59.4 Gy	11% symptomatic brainstem injury	N/A
Gunther et al., 2015 [83] Single-institution retrospective	Photon	35 children Median age 6.9 years, ependymoma	40.6 months	Median dose 54 Gy	9% symptomatic brainstem injury	N/A
Nanda et al., 2017 [77] Single-institution retrospective	Photon	60 children Median age 6.2 years IF tumors	2.8 years	CSI 18–36 Gy, Boost 54–59.4 Gy	23% for any brainstem toxicity, 3% for grade 3+	N/A
Devine et al., 2019 [84] Multi-institution retrospective	Photon	107 children Median age 8.3 years IF tumors	4.7 years	Median CSI 23.4 Gy, boost 55.8 Gy. Median brainstem Dmax 57.6 Gy	1.9% for any brainstem toxicity	N/A

IF: infratentorial; CSI: craniospinal irradiation.

**Table 2:** A summary of selected studies for children with NGGCT.

	SIOP-GCT-96 [148]	ACNS0122 [149]	ACNS1123 [147]	ACNS2021
RT field	Focal RT	CSI	WVRT	WVRT + Spinal RT
RT field prescription dose	54 Gy	36 Gy	30.6 Gy	30.6 Gy
Boost	-	Focal RT	Focal RT	Focal RT
Boost volume total dose	-	54 Gy	54 Gy	54 Gy
Timepoint	5 years	5 years	3 years	Accruing as of January 2025
PFS	72%	84%/92% *	77%/88% **	
OS	82%	93%/98% *	88%/92% **	

* All patients/patients in complete or partial response only. ** All patients/patients eligible for the therapeutic de-escalation protocol only.

**Table 3:** A summary of selected studies on the hippocampal dose and cognitive outcome for pediatric CNS tumors.

Author and Study Type	Patient Population	Median Follow Up	Radiotherapy Technique	Frequency of Neurocognitive Assessment	Hippocampal Dosimetric Outcome
Archarya et al., 2019 [158] Prospective longitudinal observation study	80 patients from 6–21 years old with low grade glioma	9.8 years	54 Gy 3D conformal or intensity modulated radiotherapy	Baseline, 6-month, yearly through year 5, year 7 or 8, then at 10 years	Volume receiving 40 Gy or higher to the right of left hippocampus associated with short-delay recall.
Goda et al., 2020 [159] Prospective longitudinal observation study	48 patients less than 13 years old with benign or low-grade tumors	5 years	54 Gy stereotactic conformal radiotherapy	Baseline, 6- month, then annually	Age < 13 years and mean left hippocampal dose > 30 Gy associated with worse FSIQ Mean left hippocampus dose > 25 Gy associated with >10% of the PQ subdomain
Zureick et al., 2018 [156] Retrospective review	70 patients less than 22 years old with primary brain tumor	3 years	Variable dose using proton therapy	Not specified	Volume receiving 20 Gy or higher to the left hippocampus associated with decline in delayed and immediate verbal and visual memory, FSIQ
Merchant et al., 2014 [160] Prospective longitudinal observation study	58 patients less than 21 years old with medulloblastoma	Not specified (upto 5 years)	23.4 Gy CSI 55.8 Gy primary site for average risk 36–39.6 Gy CSI 55.8 Gy primary site for high risk	Baseline then annually	Higher left or right hippocampi associated with lower IQ, WIAT reading, spelling, math
Redmond et al., 2013 [161] Prospective longitudinal observation study	19 patients aged 1–18 years with various brain tumors	Not specified (upto 26 months)	12 Gy–59.4 Gy using various techniques	Baseline, 6-month, 15-month, 27-month	Increasing mean dose to left or right hippocampus associated with worse motor speed and dexterity but not verbal learning or memory
Tsang et al., 2020 [157] Prospectively collected retrospective study	56 patients age 1–17 years old with various brain tumros	3.2 years	23.4 Gy–59.4 Gy using photon	Baseline and then every 2–3 years	Dose to 50% of left or right hippocampus associated with lower verbal comprehension index

FSIQ: full-scale intelligence quotient; PQ: performance quotient; WIAT: Wechsler Individual Achievement Test.

## Glossary

Bragg Peak	A phenomenon in proton therapy where the radiation dose is deposited at a specific depth, minimizing exposure to surrounding healthy tissue
Craniospinal Irradiation (CSI)	A radiation volume that encompasses the entire brain, spinal cord, and cerebrospinal fluid
CyberKnife^®^	A frameless stereotactic radiosurgery (SRS) system utilizing linear accelerator and robotic arm
Flash Radiotherapy (FLASH-RT)	An emerging high-dose, ultra-fast radiation therapy technique that aims to minimize normal tissue damage while effectively treating tumors
Fractionated Stereotactic Radiotherapy (FSRT)	A precise stereotactic radiation delivered over multiple treatment sessions
Gamma Knife^®^	A dedicated stereotactic radiosurgery system that uses multiple cobalt-60 sources producing a narrow beam of gamma radiation
Hippocampal Avoidance (HA) Radiotherapy	A technique that reduces radiation exposure to the hippocampus to preserve cognitive function
Intensity-Modulated Proton Therapy (IMPT)	A form of proton therapy that modulates the radiation dose to better conform to the tumor shape
Intensity-Modulated Radiotherapy (IMRT)	A photon-based radiation therapy technique that modulates the radiation dose to better conform to tumor shape
Linear Accelerator (LINAC)	Radiation machine that accelerates electron to generate therapeutic photon or electron
Linear Energy Transfer (LET)	A measure of the amount of energy deposited by radiation as it travels through tissue.
Passive Scatter Proton Therapy (PSPT)	A method of delivering proton therapy where a narrow beam is scattered to create a broader treatment field
Pencil Beam Scanning (PBS)	A method of delivering proton therapy technique that uses a magnet to steer narrow beam over treatment volume
Proton Arc Therapy (PAT)	A developing technique that delivers proton radiation continuously in an arc, similar to volumetric modulated arc therapy (VMAT) with photons
Proton Beam Therapy (PBT)	A form of radiation therapy that uses protons instead of photons to deliver targeted radiation, reducing damage to surrounding normal tissues
Relative Biological Effectiveness (RBE)	A factor used to compare the effectiveness of different types of radiation in causing biological damage
Stereotactic Body Radiation Therapy (SBRT)	A precise radiation technique used for tumors outside the brain, delivering high-dose radiation in fewer fractions
Stereotactic Radiosurgery (SRS)	A high-dose, single-session radiation therapy technique used for treating small brain tumors with precision
Theranostics	A treatment approach that combines diagnostics and therapy, often using radiopharmaceuticals to both image and treat tumors
Volumetric-Modulated Arc Therapy (VMAT)	A photon-based radiation therapy technique that delivers continuous radiation while the treatment machine rotates around the patient
Whole Ventricular Radiotherapy (WVRT)	A radiation approach that targets the entire ventricular system while minimizing radiation exposure to brain parenchyma

## References

[1] Brinkman T.M., Recklitis C.J., Michel G., Grootenhuis M.A., Klosky J.L. (2018). Psychological Symptoms, Social Outcomes, Socioeconomic Attainment, and Health Behaviors Among Survivors of Childhood Cancer: Current State of the Literature. J. Clin. Oncol..

[2] Otth M., Wyss J., Scheinemann K. (2022). Long-Term Follow-Up of Pediatric CNS Tumor Survivors—A Selection of Relevant Long-Term Issues. Children.

[3] Schulte F., Brinkman T.M., Li C., Fay-McClymont T., Srivastava D.K., Ness K.K., Howell R.M., Mueller S., Wells E., Strother D. (2018). Social adjustment in adolescent survivors of pediatric central nervous system tumors: A report from the Childhood Cancer Survivor Study. Cancer.

[4] Vatner R.E., Niemierko A., Misra M., Weyman E.A., Goebel C.P., Ebb D.H., Jones R.M., Huang M.S., Mahajan A., Grosshans D.R. (2018). Endocrine Deficiency As a Function of Radiation Dose to the Hypothalamus and Pituitary in Pediatric and Young Adult Patients With Brain Tumors. J. Clin. Oncol..

[5] Paulino A.C., Lobo M., Teh B.S., Okcu M.F., South M., Butler E.B., Su J., Chintagumpala M. (2010). Ototoxicity After Intensity-Modulated Radiation Therapy and Cisplatin-Based Chemotherapy in Children With Medulloblastoma. Int. J. Radiat. Oncol..

[6] Merchant T.E., Conklin H.M., Wu S., Lustig R.H., Xiong X. (2009). Late Effects of Conformal Radiation Therapy for Pediatric Patients With Low-Grade Glioma: Prospective Evaluation of Cognitive, Endocrine, and Hearing Deficits. J. Clin. Oncol..

[7] Michalski J.M., Janss A.J., Vezina L.G., Smith K.S., Billups C.A., Burger P.C., Embry L.M., Cullen P.L., Hardy K.K., Pomeroy S.L. (2021). Children’s Oncology Group Phase III Trial of Reduced-Dose and Reduced-Volume Radiotherapy with Chemotherapy for Newly Diagnosed Average-Risk Medulloblastoma. J. Clin. Oncol..

[8] Ward E., DeSantis C., Robbins A., Kohler B., Jemal A. (2014). Childhood and adolescent cancer statistics, 2014. CA A Cancer J. Clin..

[9] Merchant T.E., Bendel A.E., Sabin N.D., Burger P.C., Shaw D.W., Chang E., Wu S., Zhou T., Eisenstat D.D., Foreman N.K. (2019). Conformal Radiation Therapy for Pediatric Ependymoma, Chemotherapy for Incompletely Resected Ependymoma, and Observation for Completely Resected, Supratentorial Ependymoma. J. Clin. Oncol..

[10] Dhall G., Grodman H., Ji L., Sands S., Gardner S., Dunkel I.J., McCowage G.B., Diez B., Allen J.C., Gopalan A. (2008). Outcome of children less than three years old at diagnosis with non-metastatic medulloblastoma treated with chemotherapy on the “Head Start” I and II protocols. Pediatr. Blood Cancer.

[11] Mynarek M., von Hoff K., Pietsch T., Ottensmeier H., Warmuth-Metz M., Bison B., Pfister S., Korshunov A., Sharma T., Jaeger N. (2020). Nonmetastatic Medulloblastoma of Early Childhood: Results From the Prospective Clinical Trial HIT-2000 and An Extended Validation Cohort. J. Clin. Oncol..

[12] Bartels U., Onar-Thomas A., Patel S.K., Shaw D., Fangusaro J., Dhall G., Souweidane M., Bhatia A., Embry L., Trask C.L. (2021). Phase II trial of response-based radiation therapy for patients with localized germinoma: A Children’s Oncology Group study. Neuro-Oncology.

[13] Depuydt T. (2018). Proton therapy technology evolution in the clinic: Impact on radiation protection. Ann. ICRP.

[14] Zhang X. (2021). A Review of the Robust Optimization Process and Advances with Monte Carlo in the Proton Therapy Management of Head and Neck Tumors. Int. J. Part. Ther..

[15] Park P.C., Zhu X.R., Lee A.K., Sahoo N., Melancon A.D., Zhang L., Dong L. (2011). A Beam-Specific Planning Target Volume (PTV) Design for Proton Therapy to Account for Setup and Range Uncertainties. Int. J. Radiat. Oncol..

[16] Pflugfelder D., Wilkens J.J., Oelfke U. (2008). Worst case optimization: A method to account for uncertainties in the optimization of intensity modulated proton therapy. Phys. Med. Biol..

[17] Bettega D., Calzolari P., Chauvel P., Courdi A., Herault J., Iborra N., Marchesini R., Massariello P., Poli G.L., Tallone L. (2000). Radiobiological studies on the 65 MeV therapeutic proton beam at Nice using human tumour cells. Int. J. Radiat. Biol..

[18] McMahon S.J., Paganetti H., Prise K.M. (2018). LET-weighted doses effectively reduce biological variability in proton radiotherapy planning. Phys. Med. Biol..

[19] Cao W., Khabazian A., Yepes P.P., Lim G., Poenisch F., Grosshans D.R., Mohan R. (2017). Linear energy transfer incorporated intensity modulated proton therapy optimization. Phys. Med. Biol..

[20] de Jong B.A., Korevaar E.W., Maring A., Werkman C.I., Scandurra D., Janssens G., Both S., Langendijk J.A. (2023). Proton arc therapy increases the benefit of proton therapy for oropharyngeal cancer patients in the model based clinic. Radiother. Oncol..

[21] Volz L., Sheng Y., Durante M., Graeff C. (2022). Considerations for Upright Particle Therapy Patient Positioning and Associated Image Guidance. Front. Oncol..

[22] Chou B., Hopper A., Elster J., Crawford J.R., McConnell K., Chang A., Mundt A.J., MacEwan I. (2021). Volumetric de-escalation and improved acute toxicity with proton craniospinal irradiation using a vertebral body-sparing technique. Pediatr. Blood Cancer.

[23] Uemura S., Demizu Y., Hasegawa D., Fujikawa T., Inoue S., Nishimura A., Tojyo R., Nakamura S., Kozaki A., Saito A. (2022). The comparison of acute toxicities associated with craniospinal irradiation between photon beam therapy and proton beam therapy in children with brain tumors. Cancer Med..

[24] Yoo G.S., Yu J.I., Cho S., Han Y., Oh Y., Lim D.H., Nam H.R., Lee J.-W., Sung K.-W., Shin H.J. (2022). Chronological Analysis of Acute Hematological Outcomes after Proton and Photon Beam Craniospinal Irradiation in Pediatric Brain Tumors. Cancer Res. Treat..

[25] Aldrich K.D., Horne V.E., Bielamowicz K., Sonabend R.Y., Scheurer M.E., Paulino A.C., Mahajan A., Chintagumpala M., Okcu M.F., Brown A.L. (2021). Comparison of hypothyroidism, growth hormone deficiency, and adrenal insufficiency following proton and photon radiotherapy in children with medulloblastoma. J. Neuro-Oncol..

[26] Liu K.X., Ioakeim-Ioannidou M., Susko M.S., Rao A.D., Yeap B.Y., Snijders A.M., Ladra M.M., Vogel J., Zaslowe-Dude C., Marcus K.J. (2021). A Multi-institutional Comparative Analysis of Proton and Photon Therapy-Induced Hematologic Toxicity in Patients With Medulloblastoma. Int. J. Radiat. Oncol..

[27] Hashimoto T., Shimizu S., Takao S., Terasaka S., Iguchi A., Kobayashi H., Mori T., Yoshimura T., Matsuo Y., Tamura M. (2019). Clinical experience of craniospinal intensity-modulated spot-scanning proton therapy using large fields for central nervous system medulloblastomas and germ cell tumors in children, adolescents, and young adults. J. Radiat. Res..

[28] Song S., Park H.J., Yoon J.H., Kim D.W., Park J., Shin D., Shin S.H., Kang H.J., Kim S.-K., Phi J.H. (2014). Proton beam therapy reduces the incidence of acute haematological and gastrointestinal toxicities associated with craniospinal irradiation in pediatric brain tumors. Acta Oncol..

[29] DeLaney T.F., Liebsch N.J., Pedlow F.X., Adams J., Dean S., Yeap B.Y., McManus P., Rosenberg A.E., Nielsen G.P., Harmon D.C. (2009). Phase II Study of High-Dose Photon/Proton Radiotherapy in the Management of Spine Sarcomas. Int. J. Radiat. Oncol..

[30] Miralbell R., Lomax A., Cella L., Schneider U. (2002). Potential reduction of the incidence of radiation-induced second cancers by using proton beams in the treatment of pediatric tumors. Int. J. Radiat. Oncol..

[31] Newhauser W.D., Fontenot J.D., Mahajan A., Kornguth D., Stovall M., Zheng Y., Taddei P.J., Mirkovic D., Mohan R., Cox J.D. (2009). The risk of developing a second cancer after receiving craniospinal proton irradiation. Phys. Med. Biol..

[32] Scorsetti M., Cozzi L., Navarria P., Fogliata A., Rossi A., Franceschini D., De Rose F., Franzese C., Carlo-Stella C., Santoro A. (2020). Intensity modulated proton therapy compared to volumetric modulated arc therapy in the irradiation of young female patients with hodgkin’s lymphoma. Assessment of risk of toxicity and secondary cancer induction. Radiat. Oncol..

[33] Lautenschlaeger S., Iancu G., Flatten V., Baumann K., Thiemer M., Dumke C., Zink K., Hauswald H., Vordermark D., Mauz-Körholz C. (2019). Advantage of proton-radiotherapy for pediatric patients and adolescents with Hodgkin’s disease. Radiat. Oncol..

[34] Sakthivel V., Ganesh K.M., McKenzie C., Boopathy R., Selvaraj J. (2019). Second malignant neoplasm risk after craniospinal irradiation in X-ray-based techniques compared to proton therapy. Australas. Phys. Eng. Sci. Med..

[35] Stokkevåg C.H., Indelicato D.J., Herfarth K., Magelssen H., Evensen M.E., Ugland M., Nordberg T., Nystad T.A., Hægeland C., Alsaker M.D. (2019). Normal tissue complication probability models in plan evaluation of children with brain tumors referred to proton therapy. Acta Oncol..

[36] Vernimmen F.J., Fredericks S., Wallace N.D., Fitzgerald A.P. (2018). Long-Term Follow-up of Patients Treated at a Single Institution Using a Passively Scattered Proton Beam; Observations Around the Occurrence of Second Malignancies. Int. J. Radiat. Oncol..

[37] Mak D.Y., Siddiqui Z., Liu Z.A., Dama H., MacDonald S.M., Wu S., Murphy E.S., Hall M.D., Malkov V., Onar-Thomas A. (2022). Photon versus proton whole ventricular radiotherapy for non-germinomatous germ cell tumors: A report from the Children’s Oncology Group. Pediatr. Blood Cancer.

[38] Lim P., Rompokos V., Bizzocchi N., Gillies C., Gosling A., Royle G., Chang Y.-C., Gaze M., Gains J. (2021). Pencil Beam Scanning Proton Therapy Case Selection for Paediatric Abdominal Neuroblastoma: Effects of Tumour Location and Bowel Gas. Clin. Oncol..

[39] Su Z., Indelicato D.J., Mailhot R.B., Bradley J.A. (2020). Impact of different treatment techniques for pediatric Ewing sarcoma of the chest wall: IMRT, 3DCPT, and IMPT with/without beam aperture. J. Appl. Clin. Med Phys..

[40] Guerreiro F., Zachiu C., Seravalli E., Ribeiro C.O., Janssens G.O., Ries M., de Senneville B.D., Maduro J.H., Brouwer C.L., Korevaar E.W. (2019). Evaluating the benefit of PBS vs. VMAT dose distributions in terms of dosimetric sparing and robustness against inter-fraction anatomical changes for pediatric abdominal tumors. Radiother. Oncol..

[41] Sheikh S., Kharouta M.Z., Pidikiti R., Damico N.J., Choi S., Dorth J.A., Mansur D.B., Machtay M.X., Yao M., Bhatt A.D. (2022). Proton Beam Therapy for Locally Advanced Head and Neck Tumors: An Analysis of Dosimetric and Clinical Outcomes. Am. J. Clin. Oncol..

[42] Bates J.E., Indelicato D.J., Morris C.G., Rotondo R.L., Bradley J.A. (2020). Visual decline in pediatric survivors of brain tumors following radiotherapy. Acta Oncol..

[43] Dell’oro M., Short M., Wilson P., Hua C.-H., Gargone M., Merchant T.E., Bezak E. (2020). Influence of Target Location, Size, and Patient Age on Normal Tissue Sparing- Proton and Photon Therapy in Paediatric Brain Tumour Patient-Specific Approach. Cancers.

[44] Gram D., Brodin N.P., Björk-Eriksson T., Nysom K., Rosenschöld P.M.A. (2023). The risk of radiation-induced neurocognitive impairment and the impact of sparing the hippocampus during pediatric proton cranial irradiation. Acta Oncol..

[45] Mash L.E., Kahalley L.S., Okcu M.F., Grosshans D.R., Paulino A.C., Stancel H., De Leon L., Wilde E., Desai N., Chu Z.D. (2023). Superior verbal learning and memory in pediatric brain tumor survivors treated with proton versus photon radiotherapy. Neuropsychology.

[46] Fjæra L.F., Indelicato D.J., Handeland A.H., Ytre-Hauge K.S., Lassen-Ramshad Y., Muren L.P., Stokkevåg C.H. (2022). A case-control study of linear energy transfer and relative biological effectiveness related to symptomatic brainstem toxicity following pediatric proton therapy. Radiother. Oncol..

[47] Lassaletta Á., Morales J.S., Valenzuela P.L., Esteso B., Kahalley L.S., Mabbott D.J., Unnikrishnan S., Panizo E., Calvo F. (2023). Neurocognitive outcomes in pediatric brain tumors after treatment with proton versus photon radiation: A systematic review and meta-analysis. World J. Pediatr..

[48] Warren E.A.H., Raghubar K.P., Cirino P.T., Child A.E., Lupo P.J., Grosshans D.R., Paulino A.C., Okcu M.F., Minard C.G., Ris M.D. (2022). Cognitive predictors of social adjustment in pediatric brain tumor survivors treated with photon versus proton radiation therapy. Pediatr. Blood Cancer.

[49] Kahalley L.S., Peterson R., Ris M.D., Janzen L., Okcu M.F., Grosshans D.R., Ramaswamy V., Paulino A.C., Hodgson D., Mahajan A. (2020). Superior Intellectual Outcomes After Proton Radiotherapy Compared with Photon Radiotherapy for Pediatric Medulloblastoma. J. Clin. Oncol..

[50] Yang C.-C., Lin S.-Y., Tseng C.-K. (2018). Maintenance of multidomain neurocognitive functions in pediatric patients after proton beam therapy: A prospective case-series study. Appl. Neuropsychol. Child.

[51] Eaton B.R., Fong G.W., Ingerski L.M., Pulsifer M.B., Goyal S., Zhang C., Weyman E.A., Esiashvili N., Klosky J.L., MacDonald T.J. (2021). Intellectual functioning among case-matched cohorts of children treated with proton or photon radiation for standard-risk medulloblastoma. Cancer.

[52] Yahya N., Manan H.A. (2020). Neurocognitive impairment following proton therapy for paediatric brain tumour: A systematic review of post-therapy assessments. Support. Care Cancer.

[53] Gross J.P., Powell S., Zelko F., Hartsell W., Goldman S., Fangusaro J., Lulla R.R., Smiley N.P., Chang J.H.-C., Gondi V. (2019). Improved neuropsychological outcomes following proton therapy relative to X-ray therapy for pediatric brain tumor patients. Neuro-Oncology.

[54] Ventura L.M., Grieco J.A., Evans C.L., Kuhlthau K.A., MacDonald S.M., Tarbell N.J., Yock T.I., Pulsifer M.B. (2017). Executive functioning, academic skills, and quality of life in pediatric patients with brain tumors post-proton radiation therapy. J. Neuro-Oncol..

[55] Antonini T.N., Ris M.D., Grosshans D.R., Mahajan A., Okcu M.F., Chintagumpala M., Paulino A., Child A.E., Orobio J., Stancel H.H. (2017). Attention, processing speed, and executive functioning in pediatric brain tumor survivors treated with proton beam radiation therapy. Radiother. Oncol..

[56] Eaton B.R., Goldberg S., Tarbell N.J., Lawell M.P., Gallotto S.L., Weyman E.A., Kuhlthau K.A., Ebb D.H., MacDonald S.M., Yock T.I. (2020). Long-term health-related quality of life in pediatric brain tumor survivors receiving proton radiotherapy at <4 years of age. Neuro-Oncology.

[57] Vázquez M., Bachmann N., Pica A., Bolsi A., De Angelis C., Lomax A.J., Weber D.C. (2022). Early outcome after craniospinal irradiation with pencil beam scanning proton therapy for children, adolescents and young adults with brain tumors. Pediatr. Blood Cancer.

[58] Peters S., Frisch S., Stock A., Merta J., Bäumer C., Blase C., Schuermann E., Tippelt S., Bison B., Frühwald M. (2022). Proton Beam Therapy for Pediatric Tumors of the Central Nervous System—Experiences of Clinical Outcome and Feasibility from the KiProReg Study. Cancers.

[59] Lim P.S., Tran S., Kroeze S.G., Pica A., Hrbacek J., Bachtiary B., Walser M., Leiser D., Lomax A.J., Weber D.C. (2020). Outcomes of adolescents and young adults treated for brain and skull base tumors with pencil beam scanning proton therapy. Pediatr. Blood Cancer.

[60] Tran S., Lim P.S., Bojaxhiu B., Teske C., Baust K., Zepter S., Kliebsch U., Timmermann B., Calaminus G., Weber D.C. (2020). Clinical outcomes and quality of life in children and adolescents with primary brain tumors treated with pencil beam scanning proton therapy. Pediatr. Blood Cancer.

[61] Sato M., Gunther J.R., Mahajan A., Jo E., Paulino A.C., Adesina A.M., Jones J.Y., Ketonen L.M., Su J.M., Okcu M.F. (2017). Progression-free survival of children with localized ependymoma treated with intensity-modulated radiation therapy or proton-beam radiation therapy. Cancer.

[62] Indelicato D.J., Bradley J.A., Rotondo R.L., Nanda R.H., Logie N., Sandler E.S., Aldana P.R., Ranalli N.J., Beier A.D., Morris C.G. (2017). Outcomes following proton therapy for pediatric ependymoma. Acta Oncol..

[63] Dalmasso C., Alapetite C., Bolle S., Goudjil F., Lusque A., Desrousseaux J., Claude L., Doyen J., Bernier-Chastagner V., Ducassou A. (2024). Brainstem toxicity after proton or photon therapy in children and young adults with localized intracranial ependymoma: A French retrospective study. Radiother. Oncol..

[64] Grewal A.S., Li Y., Fisher M.J., Minturn J., Paltin I., Belasco J., Phillips P., Kang T., Lustig R.A., Hill-Kayser C. (2019). Tumor bed proton irradiation in young children with localized medulloblastoma. Pediatr. Blood Cancer.

[65] Ruggi A., Melchionda F., Sardi I., Pavone R., Meneghello L., Kitanovski L., Zaletel L.Z., Farace P., Zucchelli M., Scagnet M. (2022). Toxicity and Clinical Results after Proton Therapy for Pediatric Medulloblastoma: A Multi-Centric Retrospective Study. Cancers.

[66] Sienna J., Kahalley L.S., Mabbott D., Grosshans D., Santiago A.T., Merchant T.E., Manzar G.S., Dama H., Hodgson D.C., Chintagumpala M. (2024). Proton therapy mediates dose reductions to brain structures associated with cognition in children with medulloblastoma. Int. J. Radiat. Oncol. Biol. Phys..

[67] Young S., Phaterpekar K., Tsang D.S., Boldt G., Bauman G.S. (2023). Proton Radiotherapy for Management of Medulloblastoma: A Systematic Review of Clinical Outcomes. Adv. Radiat. Oncol..

[68] Merchant T.E., Hoehn M.E., Khan R.B., Sabin N.D., Klimo P., Boop F.A., Wu S., Li Y., Burghen E.A., Jurbergs N. (2023). Proton therapy and limited surgery for paediatric and adolescent patients with craniopharyngioma (RT2CR): A single-arm, phase 2 study. Lancet Oncol..

[69] Jimenez R.B., Ahmed S., Johnson A., Thomas H., Depauw N., Horick N., Tansky J., Evans C.L., Pulsifer M., Ebb D. (2021). Proton Radiation Therapy for Pediatric Craniopharyngioma. Int. J. Radiat. Oncol. Biol. Phys..

[70] Brisson R.J., Indelicato D.J., Bradley J.A., Aldana P.R., Klawinski D., Morris C.G., Vega R.B.M. (2024). Long-term outcomes following proton therapy for pediatric spinal low-grade glioma. Pediatr. Blood Cancer.

[71] Indelicato D.J., Rotondo R.L., Uezono H., Sandler E.S., Aldana P.R., Ranalli N.J., Beier A.D., Morris C.G., Bradley J.A. (2019). Outcomes Following Proton Therapy for Pediatric Low-Grade Glioma. Int. J. Radiat. Oncol..

[72] Giantsoudi D., Adams J., MacDonald S.M., Paganetti H. (2016). Proton Treatment Techniques for Posterior Fossa Tumors: Consequences for Linear Energy Transfer and Dose-Volume Parameters for the Brainstem and Organs at Risk. Int. J. Radiat. Oncol..

[73] Sabin N., Merchant T., Harreld J., Patay Z., Klimo P., Qaddoumi I., Armstrong G., Wright K., Gray J., Indelicato D. (2012). Imaging Changes in Very Young Children with Brain Tumors Treated with Proton Therapy and Chemotherapy. Am. J. Neuroradiol..

[74] Indelicato D.J., Flampouri S., Rotondo R.L., Bradley J.A., Morris C.G., Aldana P.R., Sandler E., Mendenhall N.P. (2014). Incidence and dosimetric parameters of pediatric brainstem toxicity following proton therapy. Acta Oncol..

[75] Haas-Kogan D., Indelicato D., Paganetti H., Esiashvili N., Mahajan A., Yock T., Flampouri S., MacDonald S., Fouladi M., Stephen K. (2018). National Cancer Institute Workshop on Proton Therapy for Children: Considerations Regarding Brainstem Injury. Int. J. Radiat. Oncol..

[76] Gentile M.S., Yeap B.Y., Paganetti H., Goebel C.P., Gaudet D.E., Gallotto S.L., Weyman E.A., Morgan M.L., MacDonald S.M., Giantsoudi D. (2018). Brainstem Injury in Pediatric Patients With Posterior Fossa Tumors Treated With Proton Beam Therapy and Associated Dosimetric Factors. Int. J. Radiat. Oncol..

[77] Nanda R.H., Ganju R.G., Schreibmann E., Chen Z., Zhang C., Jegadeesh N., Cassidy R., Deng C., Eaton B.R., Esiashvili N. (2017). Correlation of Acute and Late Brainstem Toxicities With Dose-Volume Data for Pediatric Patients With Posterior Fossa Malignancies. Int. J. Radiat. Oncol..

[78] Bojaxhiu B., Ahlhelm F., Walser M., Placidi L., Kliebsch U., Mikroutsikos L., Morach P., Bolsi A., Lomax T., Pica A. (2018). Radiation Necrosis and White Matter Lesions in Pediatric Patients With Brain Tumors Treated With Pencil Beam Scanning Proton Therapy. Int. J. Radiat. Oncol..

[79] Giantsoudi D., Sethi R.V., Yeap B.Y., Eaton B.R., Ebb D.H., Caruso P.A., Rapalino O., Chen Y.-L.E., Adams J.A., Yock T.I. (2016). Incidence of CNS Injury for a Cohort of 111 Patients Treated With Proton Therapy for Medulloblastoma: LET and RBE Associations for Areas of Injury. Int. J. Radiat. Oncol..

[80] Vogel J., Grewal A., O’reilly S., Lustig R., Kurtz G., Minturn J.E., Shah A.C., Waanders A.J., Belasco J.B., Cole K.A. (2019). Risk of brainstem necrosis in pediatric patients with central nervous system malignancies after pencil beam scanning proton therapy. Acta Oncol..

[81] Baliga S., Gallotto S., Bajaj B., Lewy J., Weyman E., Lawell M.P., Yeap B.Y., Ebb D.E., Huang M., Caruso P. (2021). Decade-long disease, secondary malignancy, and brainstem injury outcomes in pediatric and young adult medulloblastoma patients treated with proton radiotherapy. Neuro-Oncology.

[82] Upadhyay R., Liao K., Grosshans D.R., McGovern S.L., McAleer M.F., Zaky W., Chintagumpala M.M., Mahajan A., Yeboa D.N., Paulino A.C. (2022). Quantifying the risk and dosimetric variables of symptomatic brainstem injury after proton beam radiation in pediatric brain tumors. Neuro-Oncology.

[83] Gunther J.R., Sato M., Chintagumpala M., Ketonen L., Jones J.Y., Allen P.K., Paulino A.C., Okcu M.F., Su J.M., Weinberg J. (2015). Imaging Changes in Pediatric Intracranial Ependymoma Patients Treated With Proton Beam Radiation Therapy Compared to Intensity Modulated Radiation Therapy. Int. J. Radiat. Oncol..

[84] Devine C.A., Liu K.X., Ioakeim-Ioannidou M., Susko M., Poussaint T.Y., Huisman T.A., Aboian M., Brown D., Zaslowe-Dude C., Rao A.D. (2019). Brainstem Injury in Pediatric Patients Receiving Posterior Fossa Photon Radiation. Int. J. Radiat. Oncol..

[85] MacDonald S.M., Laack N.N., Terezakis S. (2017). Humbling advances in technology: Protons, brainstem necrosis, and the self-driving car. Int. J. Radiat. Oncol. Biol. Phys..

[86] Hua C., Shulkin B.L., Indelicato D.J., Li Y., Li X., Boop F.A., Merchant T.E. (2015). Postoperative cerebral glucose metabolism in pediatric patients receiving proton therapy for craniopharyngioma. J. Neurosurg. Pediatr..

[87] Dmytriw A.A., Hadjinicolaou A., Ntolkeras G., Tamilia E., Pesce M., Berto L.F., Grant P.E., Pang E., Ahtam B. (2024). Magnetoencephalography for the pediatric population, indications, acquisition and interpretation for the clinician. Neuroradiol. J..

[88] PTCOG-Particle Therapy Co-Operative Group Facilities in Operation and Facilities Under Construction. https://www.ptcog.site/index.php/facilities-under-construction.

[89] NHS England Specialised Services Clinical Reference Group for Radiotherapy (2020). Clinical Commissioning Policy: Proton Beam Therapy for Children, Teenagers and Young Adults in the Treatment of Malignant and Non-Malignant Tumours.

[90] Cancer Australia (2023). Strategy for Proton Beam Therapy for Cancer Patients in Australia.

[91] American Society for Radiation Oncology Proton Beam Therapy Model Policy. https://www.astro.org/astro/media/astro/daily%20practice/pdfs/astropbtmodelpolicy.pdf.

[92] Cancer Care Alberta Proton Therapy Guideline Advisory Group Proton Beam Radiation Therapy Clinical Practice Guideline. https://www.albertahealthservices.ca/assets/info/hp/cancer/if-hp-cancer-guide-rt002-proton-beam-RT.pdf.

[93] Health Ontario (2021). Proton beam therapy for cancer in children and adults: A health technology assessment. Ont. Health Technol. Assess. Ser..

[94] Thomas H., Timmermann B. (2020). Paediatric proton therapy. Br. J. Radiol..

[95] Lawell M.P., Indelicato D.J., Paulino A.C., Hartsell W., Laack N.N., Ermoian R.P., Perentesis J.P., Vatner R., Perkins S., Mangona V.S. (2020). An open invitation to join the Pediatric Proton/Photon Consortium Registry to standardize data collection in pediatric radiation oncology. Br. J. Radiol..

[96] Tsang D.S., Timmerman B. (2024). Improving Access to Proton Therapy in the United States and Around the World. Int. J. Radiat. Oncol..

[97] Sahgal A., Kellett S., Ruschin M., Greenspoon J., Follwell M., Sinclair J., Islam O., Perry J. (2019). A Cancer Care Ontario Organizational Guideline for the Delivery of Stereotactic Radiosurgery for Brain Metastasis in Ontario, Canada. Pract. Radiat. Oncol..

[98] Solberg T.D., Balter J.M., Benedict S.H., Fraass B.A., Kavanagh B., Miyamoto C., Pawlicki T., Potters L., Yamada Y. (2011). Quality and safety considerations in stereotactic radiosurgery and stereotactic body radiation therapy: Executive summary. Pract. Radiat. Oncol..

[99] Schiff D., Messersmith H., Brastianos P.K., Brown P.D., Burri S., Dunn I.F., Gaspar L.E., Gondi V., Jordan J.T., Maues J. (2022). Radiation Therapy for Brain Metastases: ASCO Guideline Endorsement of ASTRO Guideline. J. Clin. Oncol..

[100] Lehrer E.J., Prabhu A.V., Sindhu K.K., Lazarev S., Ruiz-Garcia H., Peterson J.L., Beltran C., Furutani K., Schlesinger D., Sheehan J.P. (2021). Proton and Heavy Particle Intracranial Radiosurgery. Biomedicines.

[101] Schmitt D., Blanck O., Gauer T., Fix M.K., Brunner T.B., Fleckenstein J., Loutfi-Krauss B., Manser P., Werner R., Wilhelm M.L. (2020). Technological quality requirements for stereotactic radiotherapy: Expert review group consensus from the DGMP Working Group for Physics and Technology in Stereotactic Radiotherapy. Strahlenther. Onkol..

[102] Eder H.G., Leber K.A., Eustacchio S., Pendl G. (2001). The role of gamma knife radiosurgery in children. Child’s Nerv. Syst..

[103] Hodgson D.C., Goumnerova L.C., Loeffler J.S., Dutton S., Black P.M., Alexander E., Xu R., Kooy H., Silver B., Tarbell N.J. (2001). Radiosurgery in the management of pediatric brain tumors. Int. J. Radiat. Oncol..

[104] Hua C., Bass J.K., Khan R., Kun L.E., Merchant T.E. (2008). Hearing Loss After Radiotherapy for Pediatric Brain Tumors: Effect of Cochlear Dose. Int. J. Radiat. Oncol..

[105] Amendola B.E., Wolf A., Coy S.R., Amendola M.A. (2003). Role of radiosurgery in craniopharyngiomas: A preliminary report. Med Pediatr. Oncol..

[106] Epstein M.A., Packer R.J., Rorke L.B., Zimmerman R.A., Goldwein J.W., Sutton L.N., Schut L. (1992). Vascular malformation with radiation vasculopathy after treatment of chiasmatic/hypothalamic glioma. Cancer.

[107] Murphy E.S., Sahgal A., Regis J., Levivier M., Fariselli L., Gorgulho A., Ma L., Pollock B., Yomo S., Sheehan J. (2024). Pediatric cranial stereotactic radiosurgery: Meta-analysis and international stereotactic radiosurgery society practice guidelines. Neuro-Oncology.

[108] Sheth N., Chen Y., Yang J. (2012). SU-E-T-453: Optimization of Dose Gradient for Gamma Knife Radiosurgery. Med. Phys..

[109] Vincent M., Michel-Amadry G., Nigoul J.M., Beltaifa Y., Regis J. (2023). Spatial accuracy of the stereotactic Leksell^®^ Vantage head frame in comparison with the standard stereotactic Leksell^®^G Frame for Gamma-Knife. Biomed. Phys. Eng. Express.

[110] Murphy E.S., Chao S.T., Angelov L., Vogelbaum M.A., Barnett G., Jung E., Recinos V.R., Mohammadi A., Suh J.H. (2016). Radiosurgery for Pediatric Brain Tumors. Pediatr. Blood Cancer.

[111] Khaledi N., Khan R., Gräfe J.L. (2023). Historical Progress of Stereotactic Radiation Surgery. J. Med Phys..

[112] Wang J., Zheng Q., Wang Y., Wang C., Xu S., Ju Z., Pan L., Bai J., Liu Y., Qu B. (2024). Dosimetric comparison of ZAP-X, Gamma Knife, and CyberKnife stereotactic radiosurgery for single brain metastasis. BMC Cancer.

[113] Pan L., Qu B., Bai J., Huang L., Wang J., Wang C., Dai X., Weidlich G., Adler J.R. (2021). The Zap-X Radiosurgical System in the Treatment of Intracranial Tumors: A Technical Case Report. Neurosurgery.

[114] Hendricks B.K., DiDomenico J.D., Barani I.J., Barranco F.D. (2021). ZAP-X Gyroscopic Radiosurgery System: A Preliminary Analysis of Clinical Applications within a Retrospective Case Series. Ster. Funct. Neurosurg..

[115] Kano H., Su Y.-H., Wu H.-M., Simonova G., Liscak R., Cohen-Inbar O., Sheehan J.P., Meola A., Sharma M., Barnett G.H. (2018). Stereotactic Radiosurgery for Intracranial Ependymomas: An International Multicenter Study. Neurosurgery.

[116] Yoo K.H., Marianayagam N.J., Park D.J., Persad A., Zamarud A., Shaghaghian E., Tayag A., Ustrzynski L., Emrich S.C., Gu X. (2024). Stereotactic Radiosurgery for Ependymoma in Pediatric and Adult Patients: A Single-Institution Experience. Neurosurgery.

[117] Tsang D.S., Burghen E., Klimo P., Boop F.A., Ellison D.W., Merchant T.E. (2018). Outcomes After Reirradiation for Recurrent Pediatric Intracranial Ependymoma. Int. J. Radiat. Oncol..

[118] Saran F., Baumert B.G., Creak A.L., Warrington A.P., Ashley S., Traish D., Brada M. (2008). Hypofractionated stereotactic radiotherapy in the management of recurrent or residual medulloblastoma/PNET. Pediatr. Blood Cancer.

[119] Abe M., Tokumaru S., Tabuchi K., Kida Y., Takagi M., Imamura J. (2006). Stereotactic Radiation Therapy with Chemotherapy in the Management of Recurrent Medulloblastomas. Pediatr. Neurosurg..

[120] Woo C., Stea B., Lulu B., Hamilton A., Cassady J. (1997). The use of stereotactic radiosurgical boost in the treatment of medulloblastomas. Int. J. Radiat. Oncol..

[121] Somaza S.C., Kondziolka D., Lunsford D., Flickinger J.C., Bissonette D.J., Albright A.L. (1996). Early Outcomes after Stereotactic Radiosurgery for Growing Pilocytic Astrocytomas in Children. Pediatr. Neurosurg..

[122] Boëthius J., Ulfarsson E., Ráhn T., Lippitz B. (2002). Gamma knife radiosurgery for pilocytic astrocytomas. J. Neurosurg..

[123] Kano H., Niranjan A., Kondziolka D., Flickinger J.C., Pollack I.F., Jakacki R.I., Lunsford L.D. (2009). Stereotactic radiosurgery for pilocytic astrocytomas part 2: Outcomes in pediatric patients. J. Neuro-Oncol..

[124] Weintraub D., Yen C.-P., Xu Z., Savage J., Williams B., Sheehan J. (2012). Gamma Knife surgery of pediatric gliomas. J. Neurosurgery: Pediatr..

[125] Hankinson T.C., Patibandla M.R., Green A., Hemenway M., Foreman N., Handler M., Liu A.K. (2015). Hypofractionated Radiotherapy for Children With Diffuse Intrinsic Pontine Gliomas. Pediatr. Blood Cancer.

[126] Bunevicius A., Sheehan J.P. (2020). Radiosurgery for Glioblastoma. Neurosurg. Clin. N. Am..

[127] Kalapurakal J.A. (2005). Radiation therapy in the management of pediatric craniopharyngiomas—A review. Child’s Nerv. Syst..

[128] Clark A.J., Cage T.A., Aranda D., Parsa A.T., Sun P.P., Auguste K.I., Gupta N. (2012). A systematic review of the results of surgery and radiotherapy on tumor control for pediatric craniopharyngioma. Child’s Nerv. Syst..

[129] Merchant T.E., Kiehna E.N., Sanford R.A., Mulhern R.K., Thompson S.J., Wilson M.W., Lustig R.H., Kun L.E. (2002). Craniopharyngioma: The St. Jude Children’s Research Hospital experience 1984–2001. Int. J. Radiat. Oncol. Biol. Phys..

[130] Gabay S., Merchant T.E., Boop F.A., Roth J., Constantini S. (2023). Shifting Strategies in the Treatment of Pediatric Craniopharyngioma. Curr. Oncol. Rep..

[131] Friedrich C., Boekhoff S., Bischoff M., Beckhaus J., Sowithayasakul P., Calaminus G., Eveslage M., Valentini C., Bison B., Harrabi S.B. (2023). Outcome after proton beam therapy versus photon-based radiation therapy in childhood-onset craniopharyngioma patients—results of KRANIOPHARYNGEOM 2007. Front. Oncol..

[132] Tsugawa T., Kobayashi T., Hasegawa T., Iwai Y., Matsunaga S., Yamamoto M., Hayashi M., Kenai H., Kano T., Mori H. (2020). Gamma Knife Surgery for Residual or Recurrent Craniopharyngioma After Surgical Resection: A Multi-institutional Retrospective Study in Japan. Cureus.

[133] Lee C.-C., Yang H.-C., Chen C.-J., Hung Y.-C., Wu H.-M., Shiau C.-Y., Guo W.-Y., Pan D.H.-C., Chung W.-Y., Liu K.-D. (2014). Gamma Knife surgery for craniopharyngioma: Report on a 20-year experience. J. Neurosurg..

[134] Minniti G., Saran F., Traish D., Soomal R., Sardell S., Gonsalves A., Ashley S., Warrington J., Burke K., Mosleh-Shirazi A. (2006). Fractionated stereotactic conformal radiotherapy following conservative surgery in the control of craniopharyngiomas. Radiother. Oncol..

[135] Combs S.E., Thilmann C., Huber P.E., Hoess A., Debus J., Schulz-Ertner D. (2007). Achievement of long-term local control in patients with craniopharyngiomas using high precision stereotactic radiotherapy. Cancer.

[136] Selch M.T., DeSalles A.A., Wade M., Lee S.P., Solberg T.D., Wallace R.E., Ford J.M., Rubino G., Cabatan-Awang C., Withers H.R. (2002). Initial Clinical Results of Stereotactic Radiotherapy for the Treatment of Craniopharyngiomas. Technol. Cancer Res. Treat..

[137] Brown P.D., Jaeckle K., Ballman K.V., Farace E., Cerhan J.H., Anderson S.K., Carrero X.W., Barker F.G., Deming R., Burri S.H. (2016). Effect of Radiosurgery Alone vs Radiosurgery with Whole Brain Radiation Therapy on Cognitive Function in Patients with 1 to 3 Brain Metastases: A Randomized Clinical Trial. JAMA.

[138] Brown P.D., Ballman K.V., Cerhan J.H., Anderson S.K., Carrero X.W., Whitton A.C., Greenspoon J., Parney I.F., Laack N.N.I., Ashman J.B. (2017). Postoperative stereotactic radiosurgery compared with whole brain radiotherapy for resected metastatic brain disease (NCCTG N107C/CEC.3): A multicentre, randomised, controlled, phase 3 trial. Lancet Oncol..

[139] Gondi V., Bauman G., Bradfield L., Burri S.H., Cabrera A.R., Cunningham D.A., Eaton B.R., Hattangadi-Gluth J.A., Kim M.M., Kotecha R. (2022). Radiation Therapy for Brain Metastases: An ASTRO Clinical Practice Guideline. Pract. Radiat. Oncol..

[140] Redmond K.J., Gui C., Benedict S., Milano M.T., Grimm J., Vargo J.A., Soltys S.G., Yorke E., Jackson A., El Naqa I. (2021). Tumor Control Probability of Radiosurgery and Fractionated Stereotactic Radiosurgery for Brain Metastases. Int. J. Radiat. Oncol..

[141] Suki D., Abdulla R.K., Ding M., Khatua S., Sawaya R. (2014). Brain metastases in patients diagnosed with a solid primary cancer during childhood: Experience from a single referral cancer center. J. Neurosurgery: Pediatr..

[142] Howard T.P., Boyle P.J., Marcus K.J., Haas-Kogan D.A., Liu K.X. (2021). Clinical outcomes for pediatric patients receiving radiotherapy for solid tumor central nervous system metastases. Pediatr. Blood Cancer.

[143] Lo A.C., Hodgson D., Dang J., Tyldesley S., Bouffet E., Bartels U., Cheng S., Hukin J., Bedard P.L., Goddard K. (2020). Intracranial Germ Cell Tumors in Adolescents and Young Adults: A 40-Year Multi-Institutional Review of Outcomes. Int. J. Radiat. Oncol..

[144] Byun H.K., Yoon H.I., Cho J., Shim K.-W., Han J.W., Lyu C.J., Kim D.-S., Suh C.-O. (2020). Optimization of Intracranial Germinoma Treatment: Radiotherapy Alone with Reduced Volume and Dose. Int. J. Radiat. Oncol. Biol. Phys..

[145] Foo J.C., Bajin I.Y., Marushchak O., McKeown T., Bouffet E., Tsang D.S., Laperriere N., Dirks P., Drake J., Ertl-Wagner B. (2023). Time to dismiss boost? Outcomes of children with localized and metastatic germinoma. J. Neuro-Oncol..

[146] Yan M., Laperriere N., Velec M., Bartels U., Ramaswamy V., Bouffet E., Tsang D.S. (2019). Redefining Ventricular Target Volume in Germinoma: Is Inclusion of Temporal Horns Necessary?. Int. J. Radiat. Oncol. Biol. Phys..

[147] Fangusaro J., Wu S., MacDonald S., Murphy E., Shaw D., Bartels U., Khatua S., Souweidane M., Lu H.-M., Morris D. (2019). Phase II Trial of Response-Based Radiation Therapy for Patients With Localized CNS Nongerminomatous Germ Cell Tumors: A Children’s Oncology Group Study. J. Clin. Oncol..

[148] Calaminus G., Frappaz D., Kortmann R.D., Krefeld B., Saran F., Pietsch T., Vasiljevic A., Garre M.L., Ricardi U., Mann J.R. (2017). Outcome of patients with intracranial non-germinomatous germ cell tumors—lessons from the SIOP-CNS-GCT-96 trial. Neuro-Oncology.

[149] Goldman S., Bouffet E., Fisher P.G., Allen J.C., Robertson P.L., Chuba P.J., Donahue B., Kretschmar C.S., Zhou T., Buxton A.B. (2015). Phase II Trial Assessing the Ability of Neoadjuvant Chemotherapy With or Without Second-Look Surgery to Eliminate Measurable Disease for Nongerminomatous Germ Cell Tumors: A Children’s Oncology Group Study. J. Clin. Oncol..

[150] Gondi V., Tomé W.A., Mehta M.P. (2010). Why avoid the hippocampus? A comprehensive review. Radiother. Oncol..

[151] Mizumatsu S., Monje M.L., Morhardt D.R., Rola R., Palmer T., Fike J.R. (2003). Extreme sensitivity of adult neurogenesis to low doses of X-irradiation. Cancer Res..

[152] Monje M.L., Mizumatsu S., Fike J.R., Palmer T. (2002). Irradiation induces neural precursor-cell dysfunction. Nat. Med..

[153] Gondi V., Pugh S.L., Tome W.A., Caine C., Corn B., Kanner A., Rowley H., Kundapur V., DeNittis A., Greenspoon J.N. (2014). Preservation of Memory With Conformal Avoidance of the Hippocampal Neural Stem-Cell Compartment During Whole-Brain Radiotherapy for Brain Metastases (RTOG 0933): A Phase II Multi-Institutional Trial. J. Clin. Oncol..

[154] Gondi V., Tolakanahalli R., Mehta M.P., Tewatia D., Rowley H., Kuo J.S., Khuntia D., Tomé W.A. (2010). Hippocampal-Sparing Whole-Brain Radiotherapy: A “How-To” Technique Using Helical Tomotherapy and Linear Accelerator–Based Intensity-Modulated Radiotherapy. Int. J. Radiat. Oncol..

[155] Brown P.D., Gondi V., Pugh S., Tome W.A., Wefel J.S., Armstrong T.S., Bovi J.A., Robinson C., Konski A., Khuntia D. (2020). Hippocampal Avoidance During Whole-Brain Radiotherapy Plus Memantine for Patients With Brain Metastases: Phase III Trial NRG Oncology CC001. J. Clin. Oncol..

[156] Zureick A.H., Evans C.L., Niemierko A., Grieco J.A., Nichols A.J., Fullerton B.C., Hess C.B., Goebel C.P., Gallotto S.L., Weyman E.A. (2018). Left hippocampal dosimetry correlates with visual and verbal memory outcomes in survivors of pediatric brain tumors. Cancer.

[157] Tsang D.S., Kim L., Liu Z.A., Janzen L., Khandwala M., Bouffet E., Laperriere N., Dama H., Keilty D., Craig T. (2021). Intellectual changes after radiation for children with brain tumors: Which brain structures are most important?. Neuro-Oncology.

[158] Acharya S., Wu S., Ashford J.M., Tinkle C.L., Lucas J.T., Qaddoumi I., Gajjar A., Krasin M.J., Conklin H.M., Merchant T.E. (2019). Association between hippocampal dose and memory in survivors of childhood or adolescent low-grade glioma: A 10-year neurocognitive longitudinal study. Neuro-Oncology.

[159] Goda J.S., Dutta D., Krishna U., Goswami S., Kothavade V., Kannan S., Maitre M., Bano N., Gupta T., Jalali R. (2020). Hippocampal radiotherapy dose constraints for predicting long-term neurocognitive outcomes: Mature data from a prospective trial in young patients with brain tumors. Neuro-Oncology.

[160] Merchant T.E., Schreiber J.E., Wu S., Lukose R., Xiong X., Gajjar A. (2014). Critical Combinations of Radiation Dose and Volume Predict Intelligence Quotient and Academic Achievement Scores After Craniospinal Irradiation in Children With Medulloblastoma. Int. J. Radiat. Oncol. Biol. Phys..

[161] Redmond K.J., Mahone E.M., Terezakis S., Ishaq O., Ford E., McNutt T., Kleinberg L., Cohen K.J., Wharam M., Horska A. (2013). Association between radiation dose to neuronal progenitor cell niches and temporal lobes and performance on neuropsychological testing in children: A prospective study. Neuro-Oncology.

[162] Sethi R., MacDonald S. (2019). Hippocampus avoidance in pediatric patients. Neuro-Oncology.

[163] Cherlow J.M., Shaw D.W., Margraf L.R., Bowers D.C., Huang J., Fouladi M., Onar-Thomas A., Zhou T., Pollack I.F., Gajjar A. (2019). Conformal Radiation Therapy for Pediatric Patients with Low-Grade Glioma: Results from the Children’s Oncology Group Phase 2 Study ACNS0221. Int. J. Radiat. Oncol..

[164] Tensaouti F., Ducassou A., Chaltiel L., Bolle S., Muracciole X., Coche-Dequeant B., Alapetite C., Bernier V., Claude L., Supiot S. (2017). Patterns of failure after radiotherapy for pediatric patients with intracranial ependymoma. Radiother. Oncol..

[165] De B., Khakoo Y., Souweidane M.M., Dunkel I.J., Patel S.H., Gilheeney S.W., De Braganca K.C., Karajannis M.A., Wolden S.L. (2018). Patterns of relapse for children with localized intracranial ependymoma. J. Neuro-Oncol..

[166] Baliga S., Adams J.A., Bajaj B.V.M., Van Benthuysen L., Daartz J., Gallotto S.L., Lewy J.R., DeNunzio N., Weyman E.A., Lawell M.P. (2022). Patterns of failure in pediatric medulloblastoma and implications for hippocampal sparing. Cancer.

[167] Padovani L., Chapon F., André N., Boucekine M., Geoffray A., Bourdeau F., Masliah-Planchon J., Claude L., Huchet A., Laprie A. (2018). Hippocampal Sparing During Craniospinal Irradiation: What Did We Learn About the Incidence of Perihippocampus Metastases?. Int. J. Radiat. Oncol. Biol. Phys..

[168] Vozenin M.-C., Bourhis J., Durante M. (2022). Towards clinical translation of FLASH radiotherapy. Nat. Rev. Clin. Oncol..

[169] Montay-Gruel P., Acharya M.M., Jorge P.G., Petit B., Petridis I.G., Fuchs P., Leavitt R., Petersson K., Gondré M., Ollivier J. (2020). Hypofractionated FLASH-RT as an Effective Treatment against Glioblastoma that Reduces Neurocognitive Side Effects in Mice. Clin. Cancer Res..

[170] Montay-Gruel P., Acharya M.M., Petersson K., Alikhani L., Yakkala C., Allen B.D., Ollivier J., Petit B., Jorge P.G., Syage A.R. (2019). Long-term neurocognitive benefits of FLASH radiotherapy driven by reduced reactive oxygen species. Proc. Natl. Acad. Sci. USA.

[171] Montay-Gruel P., Petersson K., Jaccard M., Boivin G., Germond J.-F., Petit B., Doenlen R., Favaudon V., Bochud F., Bailat C. (2017). Irradiation in a flash: Unique sparing of memory in mice after whole brain irradiation with dose rates above 100 Gy/s. Radiother. Oncol..

[172] Simmons D.A., Lartey F.M., Schüler E., Rafat M., King G., Kim A., Ko R., Semaan S., Gonzalez S., Jenkins M. (2019). Reduced cognitive deficits after FLASH irradiation of whole mouse brain are associated with less hippocampal dendritic spine loss and neuroinflammation. Radiother. Oncol..

[173] Allen B.D., Alaghband Y., Kramár E.A., Ru N., Petit B., Grilj V., Petronek M.S., Pulliam C.F., Kim R.Y., Doan N.-L. (2022). Elucidating the neurological mechanism of the FLASH effect in juvenile mice exposed to hypofractionated radiotherapy. Neuro-Oncology.

[174] Daugherty E., Zhang Y., Xiao Z., Mascia A., Sertorio M., Woo J., McCann C., Russell K., Sharma R., Khuntia D. (2024). FLASH radiotherapy for the treatment of symptomatic bone metastases in the thorax (FAST-02): Protocol for a prospective study of a novel radiotherapy approach. Radiat. Oncol..

[175] Mascia A.E., Daugherty E.C., Zhang Y., Lee E., Xiao Z., Sertorio M., Woo J., Backus L.R., McDonald J.M., McCann C. (2023). Proton FLASH Radiotherapy for the Treatment of Symptomatic Bone Metastases: The FAST-01 Nonrandomized Trial. JAMA Oncol..

[176] Breitkreutz D.Y., Shumail M., Bush K.K., Tantawi S.G., Maxime P.G., Loo B.W. (2020). Initial Steps Towards a Clinical FLASH Radiotherapy System: Pediatric Whole Brain Irradiation with 40 MeV Electrons at FLASH Dose Rates. Radiat. Res..

[177] VIDEO: FLASH Radiotherapy ‘Transformative’ in Treating Pediatric Brain Tumors. https://www.healio.com/news/hematology-oncology/20240719/video-flash-radiotherapy-transformative-in-treating-pediatric-brain-tumors.

[178] Jo H.-J., Oh T., Lee Y.-R., Kang G.-S., Park H.-J., Ahn G.-O. (2023). FLASH Radiotherapy: A FLASHing Idea to Preserve Neurocognitive Function. Brain Tumor Res. Treat..

[179] Tolboom N., Verger A., Albert N.L., Fraioli F., Guedj E., Traub-Weidinger T., Morbelli S., Herrmann K., Zucchetta P., Plasschaert S.L. (2023). Theranostics in Neurooncology: Heading Toward New Horizons. J. Nucl. Med..

[180] Agrawal A., Rangarajan V., Shah S., Puranik A., Purandare N. (2018). MIBG (metaiodobenzylguanidine) theranostics in pediatric and adult malignancies. Br. J. Radiol..

[181] DuBois S.G., Granger M.M., Groshen S., Tsao-Wei D., Ji L., Shamirian A., Czarnecki S., Goodarzian F., Berkovich R., Shimada H. (2021). Randomized Phase II Trial of MIBG Versus MIBG, Vincristine, and Irinotecan Versus MIBG and Vorinostat for Patients With Relapsed or Refractory Neuroblastoma: A Report From NANT Consortium. J. Clin. Oncol..

[182] Souweidane M.M., Kramer K., Pandit-Taskar N., Zhou Z., Haque S., Zanzonico P., Carrasquillo J.A., Lyashchenko S.K., Thakur S.B., Donzelli M. (2018). Convection-enhanced delivery for diffuse intrinsic pontine glioma: A single-centre, dose-escalation, phase 1 trial. Lancet Oncol..

[183] Menda Y., Sue O’Dorisio M., Kao S., Khanna G., Michael S., Connolly M., Babich J., O’Dorisio T., Bushnell D., Madsen M. (2010). Phase I Trial of 90Y-DOTA0-Tyr3-Octreotide Therapy in Children and Young Adults with Refractory Solid Tumors That Express Somatostatin Receptors. J. Nucl. Med..

